# Lattice Modeling of Early-Age Behavior of Structural Concrete

**DOI:** 10.3390/ma10030231

**Published:** 2017-02-25

**Authors:** Yaming Pan, Armando Prado, Rocío Porras, Omar M. Hafez, John E. Bolander

**Affiliations:** 1 Department of Civil and Environmental Engineering, University of California, Davis, CA 95616, USA; aprado@ucdavis.edu (A.P.); omhafez@ucdavis.edu (O.M.H.); jebolander@ucdavis.edu (J.E.B.); 2 School of Civil Engineering, University of Castilla-La Mancha, 13071 Ciudad Real, Spain; Rocio.Porras@uclm.es

**Keywords:** lattice models, durability mechanics, early-age behavior, concrete, solidification theory, cement hydration

## Abstract

The susceptibility of structural concrete to early-age cracking depends on material composition, methods of processing, structural boundary conditions, and a variety of environmental factors. Computational modeling offers a means for identifying primary factors and strategies for reducing cracking potential. Herein, lattice models are shown to be adept at simulating the thermal-hygral-mechanical phenomena that influence early-age cracking. In particular, this paper presents a lattice-based approach that utilizes a model of cementitious materials hydration to control the development of concrete properties, including stiffness, strength, and creep resistance. The approach is validated and used to simulate early-age cracking in concrete bridge decks. Structural configuration plays a key role in determining the magnitude and distribution of stresses caused by volume instabilities of the concrete material. Under restrained conditions, both thermal and hygral effects are found to be primary contributors to cracking potential.

## 1. Introduction

Early-age cracking is often a root cause of premature loss of safety and serviceability of concrete structures, including reinforced concrete bridge decks and slabs. Such cracking may occur due to plastic settlement and shrinkage, prior to concrete setting, or as a consequence of volumetric changes associated with thermal, hygral, or chemical phenomena that occur after setting [1,2]. The factors that affect cracking potential can be categorized in different ways, such as the grouping presented in Table 1. These factors can be viewed as design parameters, since they are controlled (at least to some degree) during the process of design, construction, and curing of the structural components.

Many of the parameters in Table 1 influence autogenous and drying shrinkages, which are prime contributors to early-age cracking of concrete bridge decks and overlays. Control of these shrinkages, possibly through the use of shrinkage reducing admixtures (SRA), has proven to be effective in reducing cracking in such structures [3,4,5,6]. Nonetheless, other factors, acting alone or in combination, may strongly influence the probability of cracking. In particular, thermal effects due to heat of hydration and heat exchange with the environment may be important [7,8,9,10,11].

Knowledge of early-age cracking has been gained primarily through laboratory testing, often using small-scale specimens, and through the monitoring of field performance. Laboratory testing allows for the examination of multiple specimens under controlled conditions, but it only approximates the circumstances of concrete bridge decks. In particular, laboratory testing might not adequately account for the restraint mechanisms and volumetric instabilities associated with actual structural and environmental boundary conditions. On the other hand, data from the monitoring of field performance are scarce and typically provide an incomplete picture of the reasons for good or poor performance. Furthermore, the consequences of variations in material composition, construction processes, and environmental conditions are difficult to anticipate without appropriate models.

This research involves: (1) the development of a methodology for simulating the early-age behavior of structural concrete; and (2) application of the methodology to assessing early-age cracking of concrete bridge decks. The methodology relies on a simple form of lattice model [12], which has been extended to represent the salient thermal, hygral, and mechanical processes that affect the early-age behavior. A primary goal of most lattice analyses of concrete has been the simulation of fracture at the meso-scale, at which the matrix, a coarse fraction of the aggregates, and the matrix-aggregate interface are explicitly modeled [13,14,15,16,17,18,19]. Such lattice models provide a quantitive linking of meso-scale processes and structural behavior. Lattice models are also effective in performing multi-field analyses with emphases on the durability mechanics of concrete materials and structures [20,21,22,23]. The lattice-based analyses presented herein differ in that concrete is regarded as a homogeneous continuum. There is no inherent disadvantage relative to approaches based on finite element technology, which may serve the research needs equally well [24,25]. Property development of the aging concrete is based on a modeling of cementitious materials hydration. The model resolves the spatial fields of temperature, relative humidity, and displacement. From these bases, realistic connections can be made between the design parameters (including materials composition, construction practice, and environmental conditions) and properties that strongly affect susceptibility to cracking, such as strength, stiffness, and creep potential. The simple, effective representation of tensile fracture is one attribute of this modeling approach.

Validation exercises are conducted using measurements taken from small-scale laboratory tests and an instrumented bridge deck. The validated model is used to assess the various contributors to the early-age cracking potential of concrete bridge decks. Along with autogenous deformations and hygral gradients caused by the drying of exposed surfaces, thermal effects may be a prime contributor to deck cracking. The importance of restraint conditions on cracking potential is examined.

## 2. Modeling Framework

### 2.1. Program Structure

**Figure 1:**
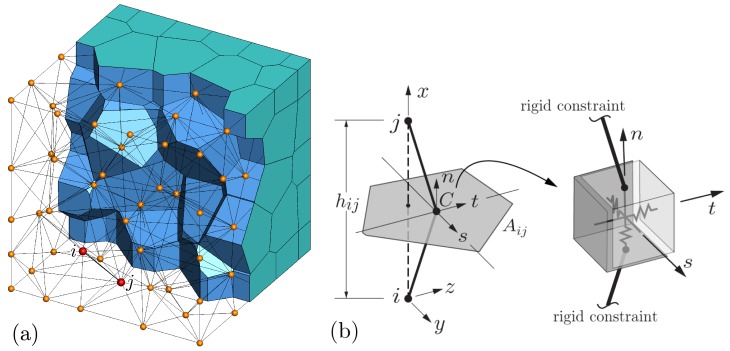
Lattice model: (**a**) domain discretization based on Delaunay and Voronoi tessellations; (**b**) lattice element i−j.

The analyses of concrete early-age behavior are based on lattice models, which are composed of randomly positioned nodal points that are interconnected by lattice elements (Figure 1). The three relevant fields (i.e., temperature, relative humidity, and displacement) are represented at the nodal points of the lattice. With respect to temperature and relative humidity analyses, the lattice elements may be viewed as conduits that transport heat or moisture between each i−j pair of nodes [21]. With respect to the structural analyses, the lattice elements are based on the rigid-body-spring concept [12,26] and are akin to conventional frame elements.

The program is driven by a modeling of cementitious materials hydration, along with the influence of environmental boundary conditions that may vary with time. As described later, the hydration model also serves to determine mechanical properties, including tensile strength, elastic modulus, and creep behavior. After each time step of the thermal-hygral-structural analyses, the incremental changes in temperature and humidity are calculated. Corresponding thermal and hygral strain increments are determined and then introduced into the structural analysis.

### 2.2. Domain Discretization

The lattice topology is defined by the Delaunay tessellation of a set of randomly placed nodes (Figure 1); the Voronoi tessellation of the same set of nodes defines element properties [21]. The discretization process is semi-automated and involves the sequential definition of Voronoi vertices, edges, and faces, followed by random filling of the domain interior with nodal points [27]. Since the vertices, edges, and faces of the body are defined by sets of four, three, and two nodes, respectively, the discretization of complex geometries is cumbersome relative to use of the finite element method.

The use of irregularly spaced nodal points, connected by 1D elements, typically introduces spurious heterogeneity into the modeling effort. Such irregular lattices can be rendered homogenous through special techniques [28] or, as done herein, by scaling the element properties according to the Voronoi tessellation of the domain [12]. As demonstrated in previous studies, this form of lattice model is also adept at representing heterogeneity, either explicitly or by using probabilistic methods [29,30]. For modeling structural concrete, however, large computational demands are incurred by the necessity of finer, three dimensional discretizations. Alternatively, lattice simulations of meso-scale heterogeneity can be used to support macro-scale analyses of homogeneous continua [8].

### 2.3. Cementitious Materials Hydration

Desirable features of the hydration model include the ability to simulate the hydration of blended cements, accounting for the chemical composition of the blend constituents. Herein, we use a versatile model of cementitious materials hydration [31,32]. Model development and calibration, done by Riding et al. [31], was based on a dataset of 204 concrete mixtures, which were tested using semi-adiabatic calorimetry. The cementitious materials considered by the model include portland cement, slag, fly ash and/or silica fume. The model has been validated through comparisons with a separate set of 57 semi-adiabatic calorimetry tests.

The influence of temperature on the rate of hydration is represented by a maturity relation

(1) $$t_{e}=\sum_{0}^{t}\;\exp\left(-\frac{E_{a}}{R}\left(\frac{1}{T_{c}}-\frac{1}{T_{r}}\right)\right)\;\Delta t$$

in which $t_{e}$ (h) is the equivalent age for a material hydrating at reference temperature $T_{r}$ (K); *R* is the universal gas constant (8.314 J/mol/K); $T_{c}$ is the temperature of the concrete (K); and $E_{a}$ is the apparent activation energy (J/mol), which depends on composition and proportioning of the cementitious materials [33].

The degree of cementitious materials hydration is assumed to be

(2) $$\alpha \left(t\right)=\frac{H(t)}{H_{u}}$$

where H(t) is the cumulative amount of heat produced by the hydration reaction (J/g); and $H_{u}$ is the total heat available for reaction (J/g), which depends on the composition and proportioning of the cementitious materials.

(3) $$H_{u}=H_{\mathrm{cem}}\;p_{\mathrm{cem}}+461\;p_{\mathrm{slag}}+1800\;p_{\mathrm{FA}-\mathrm{CaO}}\;p_{\mathrm{FA}}+330\;p_{\mathrm{SF}}$$

in which each term represents the heat of hydration of component *i* multiplied by its weight fraction, $p_{i}$. The heat of hydration of the cement (J/g) is

(4) $$\begin{array}{cc} H_{cem}= & 500\;p_{C_{3}S}+260\;p_{C_{2}S}+866\;p_{C_{3}A}+420\;p_{C_{4}\mathrm{AF}}\;+\\ & 624\;p_{{\mathrm{SO}}_{3}}+1186\;p_{\mathrm{free}\;\mathrm{Ca}}+850\;p_{\mathrm{MgO}} \end{array}$$

The relationship between degree of hydration and equivalent age is

(5) $$\alpha \left(t_{e}\right)=\alpha_{u}\;\exp\;\left(-{\left[\frac{\tau }{t_{e}}\right]}^{\beta }\right)$$

in which *β* and *τ* are hydration parameters; and $\alpha_{u}$ is the ultimate degree of cementitious materials reaction. The rate of heat release with respect to time is calculated by combining the information presented in Equations (1), (2) and (5).

(6) $$Q\left(t\right)=H_{u}c{\left(\frac{\tau }{t_{e}}\right)}^{\beta }\left(\frac{\beta }{t_{e}}\right)\;\alpha_{u}\;\exp\;\left(-{\left[\frac{\tau }{t_{e}}\right]}^{\beta }\right)\;\exp\;\left(\frac{E_{a}}{R}\left(\frac{1}{T_{r}}+\frac{1}{T_{c}}\right)\right)$$

where *c* is the cementitious materials content (g/m${}^{3}$). The rate of heat release depends on *β*, *τ*, and $\alpha_{u}$, which themselves depend on the composition and proportioning of the cementitious materials. These dependencies have been established through multi-variate regression analyses based on concrete mixtures with a wide range of compositions [31].

### 2.4. Primary Analysis Modules

#### 2.4.1. Thermal Analysis

The governing differential equation for heat conduction is

(7) $$\nabla \;\cdot \;\left(\lambda \nabla T\right)+\rho c_{p}\;\frac{Q}{c_{p}}=\rho c_{p}\frac{\partial T}{\partial t}$$

in which *T* is temperature (K), *Q* is the rate of heat production (J/(kg · s)), *λ* is thermal conductivity (W/(m · K)), *ρ* is mass density (kg/m${}^{3}$), and $c_{p}$ is specific heat capacity (J/(kg · K)). The boundary conditions for Equation (7) involve either prescribed temperatures or heat flux across the boundary. For bridge deck systems, heat exchange with the environment is of primary importance. Herein, the following types of heat exchange are modeled:

- Convection—Convective heat exchange across exposed surfaces depends on the difference between the solid surface temperature $T_{s}$ and that of the surrounding ambient medium $T_{a}$
  (8) $$q_{conv}=\Lambda_{T}\left(T_{s}-T_{a}\right)$$
  where $\Lambda_{T}$ is the coefficient of convective heat transfer, which depends on wind speed and other factors [34].
- Solar radiation—The amount of solar radiation reaching the concrete surface depends on several factors including the structure’s location, surface orientation, altitude, atmospheric conditions, time of the day, and day of the year. Incoming heat due to solar radiation is
  (9) $$q_{sun}=\gamma_{abs}q_{inc}$$
  in which $\gamma_{abs}$ is the solar absorptivity of the concrete, which is influenced by the color and texture of the concrete surface, and $q_{inc}$ is the incident solar radiation acting on a horizontal surface (W/m${}^{2}$). The latter of the two values can be obtained from recorded weather data for the time period and region of interest.
- Thermal radiation - Heat loss to the surroundings due to grey-body radiation is calculated using
  (10) $$q_{sky}=\sigma \epsilon \left(T_{sK}^{4}-T_{sky}^{4}\right)$$
  where *σ* is the Stefan-Boltzmann constant ($5.669\times 10^{-2}$ W/(m${}^{2}$ · K${}^{4}$)), *ϵ* is the emissivity of the concrete (=0.9, in this study), and $T_{sK}$ is the temperature of the concrete surface (K). $T_{sky}$ is the sky temperature, which depends on the sky emissivity, the dew point temperature, and the cloud conditions [34].

Heat exchange also occurs due to evaporation and condensation, but those mechanisms are considered to be of secondary importance for ordinary concrete structures. Some other relevant aspects of the thermal analyses are as follows.

- The heat capacity of the cement paste is estimated using an approach given by Bentz [35], in which heat capacity is a function of degree of reaction of the cement. The heat capacity of the concrete is then determined from the heat capacities of the cement paste and aggregates, according to the mass fractions of each using an ordinary rule of mixtures.
- Thermal conductivity of the concrete is estimated by taking the average of the Hashin-Shtrikman bounds for a two-phase composite formed of paste and aggregates [35].

For the lattice modeling of heat conduction, the material continuum is represented by a collection of 1D elements connected on a semi-random set of nodal points (Figure 1). Each edge of the Delaunay tessellation, connecting adjacent nodes *i* and *j*, acts as a lineal conduit element. A semi-discrete form of Equation (7) serves as the basis for this lattice modeling of heat conduction.

(11) $$M\dot{T}+KT=f$$

where the capacity, M, and conductivity, K, matrices are assembled from their respective elemental contributions:

(12) $$M_{e}=\frac{h_{ij}A_{ij}}{6d}\left[\begin{array}{cc} 2 & 1\\ 1 & 2 \end{array}\right],$$

(13) $$K_{e}=D\frac{A_{ij}}{h_{ij}}\left[\begin{array}{cc} 1 & -1\\ -1 & 1 \end{array}\right]$$

in which $A_{ij}$ is the area of the Voronoi facet associated with nodes *i* and *j*, $h_{ij}$ is the distance between the nodes, and *D* is thermal diffusivity (= $\lambda /\rho c_{p}$). In the element capacity matrix, *d* = 1, 2, and 3 for 1D, 2D, and 3D networks, respectively. For each time step, Equation (11) is solved using the Crank-Nicholson method [36] in conjunction with a fixed-point algorithm [37] to achieve convergence. That is, Equation (11) is solved and corrected (for the dependencies of *Q* on *T* and $c_{p}$ on *α*) until the $L^{2}$ norm of the difference of nodal temperature values between iterations is satisfactorily small.

Thermal strains in the elastic material arise from changes in temperature at the lattice nodes:

(14) $$\Delta \varepsilon^{T}=\beta_{T}\Delta T$$

where $\beta_{T}$ is the coefficient of thermal expansion (CTE) and ΔT is the difference in nodal temperature over the time step. Equation (14) uses the average of ΔT calculated at the nodes of a given element; $\Delta \varepsilon^{T}$ is then introduced in the axial direction of the corresponding element.

**Figure 2:**
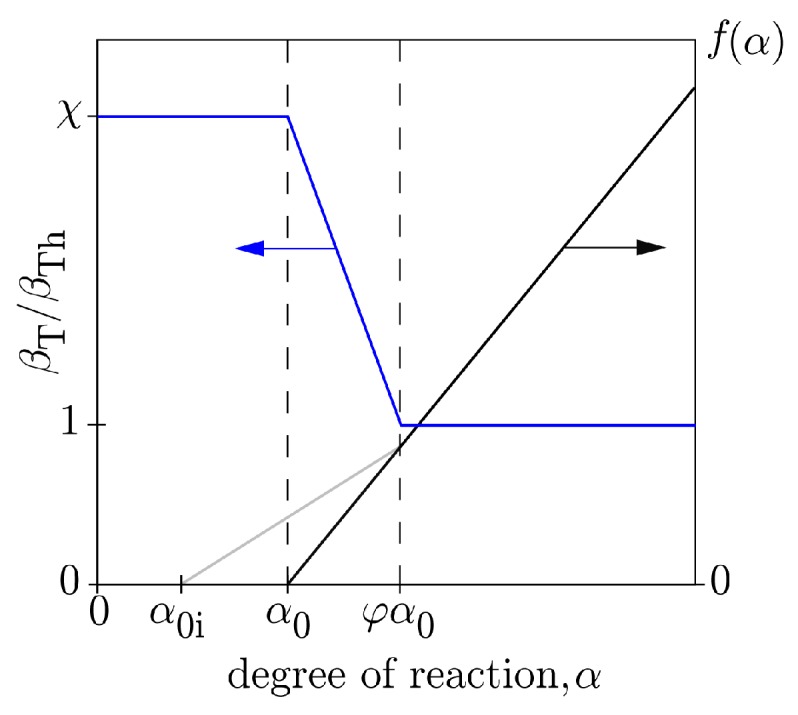
Assumed relationship between coefficient of thermal expansion and degree of cementitious materials reaction.

The value of $\beta_{T}$ depends on several factors, including moisture content and the type of aggregate. The dependence on moisture content is particularly strong, such that $\beta_{T}$ is significantly larger during the setting process and for several hours thereafter [38,39,40]. To account for this behavior, the coefficient of thermal expansion is modeled as indicated in Table 2, in which $\alpha_{Th}$ is the CTE of hardened concrete and *χ* represents the magnification of CTE prior to final setting. The relationship between $\beta_{T}$, degree of reaction *α*, and the setting parameters ($\alpha_{0i}$, $\alpha_{0}$, and φ, as described by Krauß and Rostásy [41]) is shown in Figure 2.

#### 2.4.2. Hygral Analysis

The moisture field within the concrete varies with time due to both autogenous and external drying effects. Using relative humidity *h* as the field variable [42,43], the governing differential equation can be expressed as

(15) $$\frac{\partial h}{\partial t}=\mathrm{div}\left(D_{h}\left(h\right)\mathrm{grad}h\right)+\frac{\partial h_{s}}{\partial t}$$

where $D_{h}$ is hygral diffusivity and $h_{s}$ is relative humidity associated with self-desiccation. Herein, $h_{s}$ is assumed to be a function of degree of hydration [44]

(16) $$h_{s}=\left(h_{su}-1\right){\left(\frac{\alpha }{\alpha_{u}}\right)}^{s}+1$$

where $h_{su}$ is the asymptotic value of relative humidity as $\alpha /\alpha_{u}$ approaches unity under sealed conditions. Parameter *s* controls the rate of humidity drop and is thought to depend on w/c [44].

**Figure 3:**
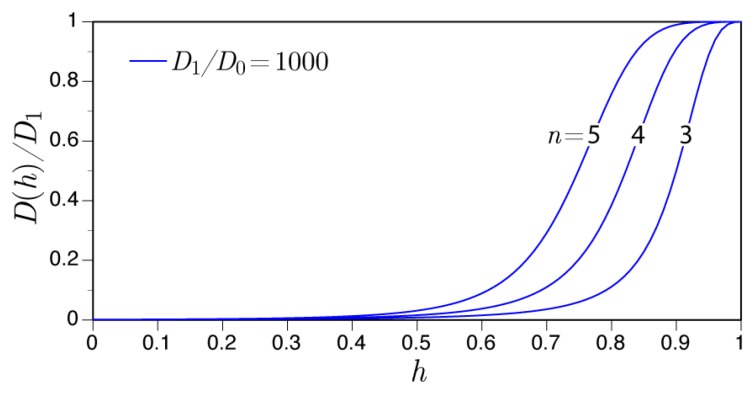
Dependence of hygral diffusivity on relative humidity according to Equation (17) with ψ(T) = 1.

Various expressions have been used to represent the dependence of hygral diffusivity on moisture content of the material. Herein, the expression of di Luzio and Cusatis is employed [45].

(17) $$D_{h}\left(h,T\right)=\psi \left(T\right)D_{1}{\left[1+\left(\frac{D_{1}}{D_{0}}-1\right){\left(1-h\right)}^{n}\right]}^{-1}$$

where

(18) $$\psi \left(T\right)=\exp\left(\frac{E_{a}}{R}\left(\frac{1}{T_{0}}-\frac{1}{T}\right)\right),$$

which represents the influence of temperature on diffusivity. $D_{1}$ (m${}^{2}$/h) is the diffusivity in the saturated state (*h* = 1), $D_{0}$ (m${}^{2}$/h) is the diffusivity of the fully dried material (*h* = 0), and $T_{0}$ is the reference temperature (K). Parameter *n* controls the position of the diffusivity drop as the concrete dries from a saturated state (Figure 3). The temperature dependence of $D_{h}$ is deemed to be important, since concrete bridge decks may experience significant solar heating during the day and cooling at night. For a portland cement concrete, the dependence of diffusivity on water-to-cement ratio, w/c, can be represented by [45]

(19) $$D_{0}=\tilde{D_{0}}{\left(\frac{w}{c}\right)}^{3}\;\;\mathrm{and}\;\;D_{1}=\tilde{D_{1}}{\left(\frac{w}{c}\right)}^{2.5}$$

where $\tilde{D_{0}}$ (m${}^{2}$/h) and $\tilde{D_{1}}$ (m${}^{2}$/h) are material parameters.

Convective transport of moisture across exposed surfaces is governed by

(20) $$q_{h}=\Lambda_{h}\left(h-h_{a}\right)$$

in which $\Lambda_{h}$ is the hygral convection coefficient and $h_{a}$ is the ambient relative humidity.

The lattice representation of the hygral field, and its solution procedure, are analogous to that of the thermal field (i.e., the lattice elements can be viewed as conduits that transfer moisture between nodes of differing relative humidity). This approach was developed by Sadouki and van Mier [46] and later adapted to this form of random lattice [21,23]. Alternatively, the hygral field can be represented by a dual lattice network defined by the Voronoi edges of the tessellated volume [47,48,49]. The main advantage of this alternative approach comes in the post-cracking state: elements are aligned with the crack surfaces, which more naturally accounts for crack-assisted transport and diffusion from the crack faces into the bulk material.

At this stage of model development, it is assumed that the diffusion process is uncoupled from the mechanical behavior of the material and any damage incurred during mechanical or hygral loading. Shrinkage strains in the elastic material arise from changes in relative humidity at the lattice nodes. A constant hygral shrinkage coefficient $\beta_{h}$ is used in this one-way coupling of the hygral and mechanical analyses

(21) $$\Delta \varepsilon^{h}=\beta_{h}\Delta h$$

where Δh is the difference in nodal relative humidity over the time step. Equation (21) uses the average of Δh calculated at the nodes of a given element and $\Delta \varepsilon^{h}$ is then introduced in the axial direction of the corresponding element.

Coupling of the thermal and hygral fields is not considered herein, but has been included in other studies [50,51]. Such coupling is particularly relevant when simulating moderate to high-temperature loading of concrete [52,53].

#### 2.4.3. Structural Analysis

A structural element is defined by two neighboring nodes, *i* and *j*, and their common Voronoi facet (Figure 1). The element stiffness relations are based on a zero-size spring set, located at the area centroid (point *C*) of the Voronoi facet, and connected to the element nodes via rigid-arm constraints. The spring set consists of three axial springs, oriented normal and tangential to the facet, and three rotational springs about the same local (*n*-*s*-*t*) axes. The stiffness coefficients of the axial springs are:

(22) $$k_{n}=\xi k_{s}=\xi k_{t}=E\frac{A_{ij}}{h_{ij}},$$

where $A_{ij}$ is the Voronoi facet area; $h_{ij}$ is the distance between nodes *i* and *j*; factor *ξ* relates the normal and shear spring stiffnesses, which can be adjusted to simulate macroscopic Poisson ratio of the material [18]. In most cases of random discretization, there will be boundary layer effects on the mechanical properties [54]. For *ξ* = 1, which is used herein, the lattice is elastically homogeneous under uniform modes of straining [55,56], although Poisson ratio *ν* = 0. Correct representation of the Poisson effect, both macroscopically and in an element-local sense, has recently been accomplished using an iterative procedure [57,58]. The stiffness coefficients of the rotational springs are:

(23) $$k_{\phi n}=E\frac{J_{p}}{h_{ij}},\;\;\;k_{\phi s}=E\frac{I_{11}}{h_{ij}},\;\;\;k_{\phi t}=E\frac{I_{22}}{h_{ij}},$$

where $J_{p}$ is the polar second moment of the facet area, and $I_{11}$ and $I_{22}$ are the two principal second moments of the facet area. Directions *s* and *t* are aligned with the facet principal axes. The spring constants appear on the diagonal of the material matrix, D, given by

(24) $$D=\left(1-\omega \right)\;\mathrm{diag}\left[k_{n},\;k_{s},\;k_{t},\;k_{\phi n},\;k_{\phi s},\;k_{\phi t}\right],$$

where *ω* is a scalar damage parameter used to model material fracture. Prior to fracture initiation, *ω* = 0. The element stiffness matrix (with respect to element local coordinates) is

(25) $$K_{e}=B^{T}DB.$$

in which B relates the generalized spring displacements and nodal displacements [55]. After transforming $K_{e}$ to global coordinates, the direct stiffness approach is used to assemble element stiffness matrices and internal force contributions into the structural equation set

(26) KΔu=(R+F)

in which Δu is the increment in generalized nodal displacements; K is the system stiffness matrix assembled from the elemental components; R and F are the external and internal nodal force vectors, respectively.

**Figure 4:**
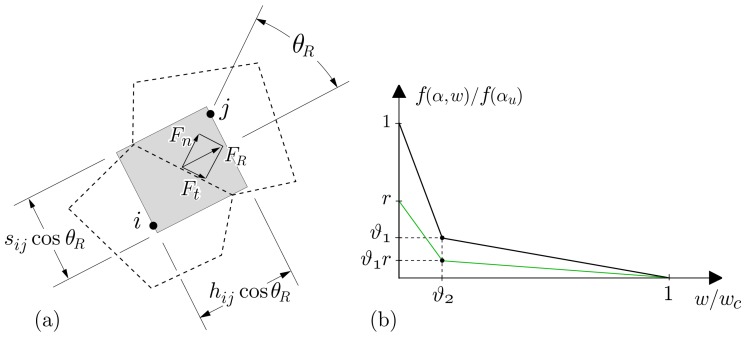
(**a**) Determination of tensile stress for planar analyses; and (**b**) tension softening relation. Parameters $\vartheta_{1}$ and $\vartheta_{2}$ define the break point of the softening diagram in terms of tensile strength $f(\alpha_{u})$ and traction-free crack opening $w_{c}$, respectively.

After each equilibrium iteration within a load increment, the spring set forces of each element ij are updated based on Δu and the spring stiffnesses (Equations (22) and (23)), which are related through the rigid-body constraints. The tensile stress resultant acting in a given element is

(27) $$\sigma_{R}=\frac{\sqrt{F_{n}^{2}+F_{s}^{2}+F_{t}^{2}}}{A_{ij}^{P}}$$

where $F_{n}$, $F_{s}$, and $F_{t}$ are the forces in the normal and two tangential springs, respectively; and $A_{ij}^{P}$ is the projection of the Voronoi facet area of element ij on a plane perpendicular to the force resultant. A planar representation of this concept is shown in Figure 4a, where $\theta_{R}$ indicates the inclination of the force resultant relative to the element axis; and $s_{ij}$ is the length of the Voronoi segment common to nodes *i* and *j*, such that $A_{ij}^{P}$ = $bs_{ij}\cos\theta_{R}$ where *b* is the element thickness. The dimension of $h_{ij}\cos\theta_{R}$ represents the crack band width [59].

Tensile fracture is simulated using an event-by-event approach [19,60], in which the element with the largest stress ratio $\kappa =\max\left[\sigma_{R}/f\left(\alpha ,w\right)\right]\geq 1$ undergoes fracture. At degree of hydration *α*, residual tensile strength f(α,w) is defined by a softening relation in terms of crack opening *w* (Figure 4b). This approach provides energy conserving, grid-insensitive simulations of tensile fracture, as confirmed by previous studies [48,55,56].

The spring set properties evolve with degree of cementitious materials hydration. Creep and stiffness development are based on solidification and microprestress theory, as described in the next section. The development of tensile strength is represented by

(28) $$f\left(\alpha \right)=f\left(\alpha_{u}\right){\left(\frac{\alpha -\alpha_{0}}{\alpha_{u}-\alpha_{0}}\right)}^{\zeta }\;\;\;\mathrm{for}\;\;\;\alpha \geq \alpha_{0}$$

where $f(\alpha_{u})$ is the tensile strength at the ultimate degree of hydration; $\alpha_{0}$ is the degree of hydration associated with setting; and coefficient *ζ* =1 when tensile strength is being modeled [41,61]. The softening relation evolves as a function of degree of hydration, as shown in Figure 4b where *r* is the fraction of ultimate tensile strength acting at hydration degree, *α*. By holding $w_{c}$ constant, fracture energy (i.e., the area under the softening curve) grows with degree of hydration, whereas the characteristic length decreases [62,63,64]. Kinetics of the reaction are also influential and can be modeled [25,65], but are neglected herein for the sake of simplicity and in light of the uncertainties in the model inputs. In Equation (28), strength development depends phenomenologically on the degree of hydration. Alternatively, property development can be based on explicit representations of the cementitious materials and their hydration products [66,67,68]. The increased physical bases of such microstructural models are attractive, especially when simulating early-age behavior.

If *f* is based on a short duration tensile test, cracking occurs at $\sigma_{R}/f<1$ under sustained loading due the occurrence of tertiary creep. From uniaxial tension tests, Domone [69] found that creep rupture occurs when the sustained stress exceeds 0.75 *f*. Altoubat and Lange [70] measured the fraction of tensile strength acting at the time of cracking to be about 0.60 to 0.64 for split cylinder tests, and 0.75 to 0.80 for uniaxial tension tests. van Breugel found that, under longer duration loading caused by self-induced stress, early-age cracking occurs at about 0.75 *f* [71]. From beam tests, Wittmann et al. [72] determined the stress at failure to be less than the tensile strength (measured by a short duration test) by a factor of 0.6 to 0.8. Emborg [7,62] reports that for low loading rates, such as those associated with the cooling of mass concrete, the reduction factor applied to tensile strength is about 0.7. A reduction factor of 0.7 is assumed for the parametric analyses presented in Section 4.

### 2.5. Stiffness and Creep Representation

**Figure 5:**
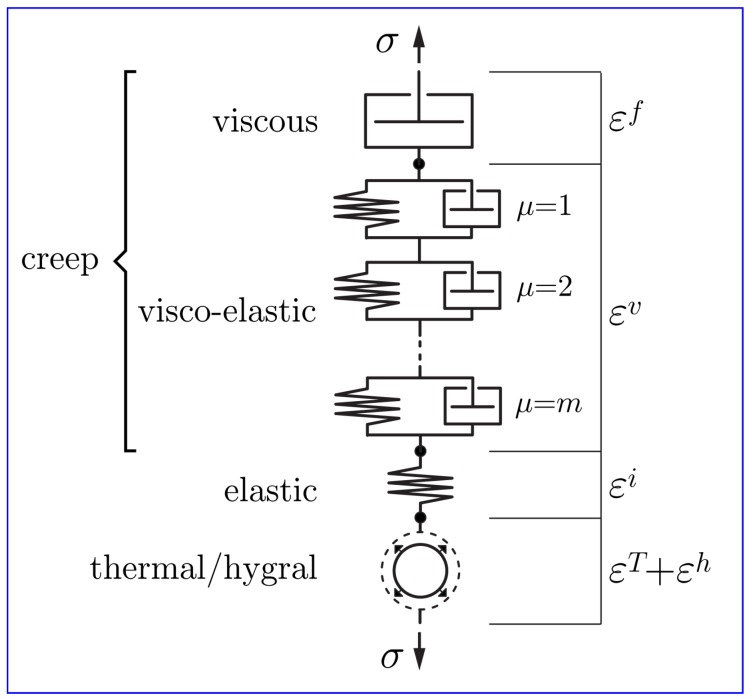
Series construction of total strain components.

The stiffness and creep of concrete are modeled using solidification and microprestress theory [25,73,74]. In effect, the axial (*n*-component) springs of the rigid-body-spring network have been replaced with the series construct shown in Figure 5. The construct consists of: (1) a spring representing instantaneous elastic deformation, $\varepsilon^{i}$; (2) a chain of *m* solidifying Kelvin units representing viscoelastic creep, $\varepsilon^{v}$; (3) an aging dashpot with viscosity that is dependent on microprestress, representing viscous creep, $\varepsilon^{f}$; and 4) a unit representing the combination of thermal strain, $\varepsilon^{T}$, and hygral strain, $\varepsilon^{h}$. The shear (*s*- and *t*-component) springs of the rigid-body-spring network are replaced by the same construct, but without the thermal and hygral unit. Superposition of the individual strain units, and differentiating their values with respect to time, produces the total strain rate:

(29) $$\dot{\varepsilon }={\dot{\varepsilon }}^{i}+{\dot{\varepsilon }}^{v}+{\dot{\varepsilon }}^{f}+{\dot{\varepsilon }}^{h}+{\dot{\varepsilon }}^{T}$$

The relationship between instantaneous strain rate and uniaxial stress rate, $\dot{\sigma }$, can be expressed by

(30) $${\dot{\varepsilon }}^{i}=q_{1}\dot{\sigma }$$

where $q_{1}$ (MPa${}^{-1}$) is an age independent parameter.

According to solidification theory, viscoelastic strains arise from creep of the solidified hydration products (which are mainly calcium-silicate-hydrate gels) whose properties do not change with age. Viscoelastic microstrain has the following formulation:

(31) $$\gamma =\int_{0}^{t}\Phi \left(t_{r}\left(t\right)-t_{r}\left(\tau \right)\right)\;\dot{\sigma }d\tau$$

in which $t_{r}\left(t\right)$ is a reduced time function that accounts for the effects of temperature and humidity on fine-scale creep processes; $\Phi (t-t_{0})$ represents the non-aging micro-compliance function of cement gel, which can be defined as

(32) $$\Phi \left(t-t_{0}\right)=q_{2}\ln\left[1+{\left(t-t_{0}\right)}^{0.1}\right]$$

where $t-t_{0}$ is the load duration, and $q_{2}$ (MPa${}^{-1}$) is an adjustable model parameter [73].

Aging of the cement paste matrix is attributed to accumulation of the solidified hydration products. The viscoelastic micro- and macro-strain rates are related as follows:

(33) $${\dot{\varepsilon }}^{v}\left(t\right)=\frac{1}{v(\alpha )}\dot{\gamma }$$

where v(α) is an aging function, representing the volume fraction of solidified material at degree of hydration, *α*. Following di Luzio and Cusatis [25], the aging function is expressed as:

(34) $$v\left(\alpha \right)={\left[\frac{\alpha }{\alpha_{u}}\right]}^{n_{\alpha }}$$

where $n_{\alpha }$ is an adjustable model parameter.

The determination of viscoelastic microstrains, *γ*, using the history integral formulation of Equation (31) is computationally expensive, even for modest numbers of degrees of freedom. The problem is therefore recast as a rate formulation based on a Dirichlet series expansion of $\Phi (t-t_{0})$. Details regarding this approximation, the determination of viscoelastic compliance, and other aspects of the numerical implementation of the viscoelastic creep model are given elsewhere [25,73].

According to the microprestress theory [73], hindered adsorbed water produces a disjoining pressure within the micropores, which induces a microprestress in the solidified material. For modeling purposes, microprestress *S* is defined as the average stress acting on bonds that bridge the micropores. Viscous creep results from shear slip caused by the breakage of overstressed bonds. Under varying temperature and humidity conditions, the viscous creep flow is

(35) $${\dot{\varepsilon }}^{f}\left(t\right)=\psi \left(t\right)\frac{\sigma (t)}{\eta \left(S\right)}$$

where the viscosity *η* is a decreasing function of the microprestress, which is given by

(36) $$\frac{1}{\eta \left(S\right)}=q_{4}\kappa_{0}S$$

where $q_{4}$ (MPa${}^{-1}$) and $\kappa_{0}$ are adjustable parameters [25]. The evolution of microprestress is governed by

(37) $$\dot{S}+\psi_{S}\kappa_{0}S^{2}=\kappa_{1}\left|\dot{T}\ln\left(h\right)+T\frac{\dot{h}}{h}\right|$$

where $\kappa_{1}$ is a model parameter. In the above formulation, *ψ* and $\psi_{S}$ are reduced time coefficients representing the effects of temperature and relative humidity on the creep processes and microprestress evolution, respectively. The coefficient applied to the creep processes is

(38) $$\psi =\left(0.1+0.9h^{2}\right)\exp\left[\frac{E_{v}}{R}\left(\frac{1}{T_{0}}-\frac{1}{T\left(t\right)}\right)\right]$$

where $E_{v}$ is the activation energy ($E_{v}/R\approx 5000$ K). The coefficient applied to microprestress evolution, $\psi_{S}$, takes the same form except that activation energy $E_{S}$ replaces $E_{v}$ ($E_{S}/R\approx 3000$ K). Microprestress can be determined from a central difference approximation of Equation (37). Details of this determination, and other aspects of the viscous creep modeling, are provided elsewhere [25,73].

## 3. Validation Exercises

### 3.1. Stiffness and Basic Creep Development

The implementation of solidification and microprestress theory [25,74] is verified for the case of basic creep. Although numerous creep test data are available for comparison, cases of early age loading are of primary interest herein. The results of Laplante [75] have been commonly used as a benchmark for creep behavior beginning with loading at 24 h [25,65].

Cylindrical specimens were loaded at 1, 3, 7, and 28 days after casting [75]. The specimens were sealed to prevent moisture exchange between the concrete and environment. The specimens were loaded at 30% of the concrete strength at each respective age.

**Figure 6:**
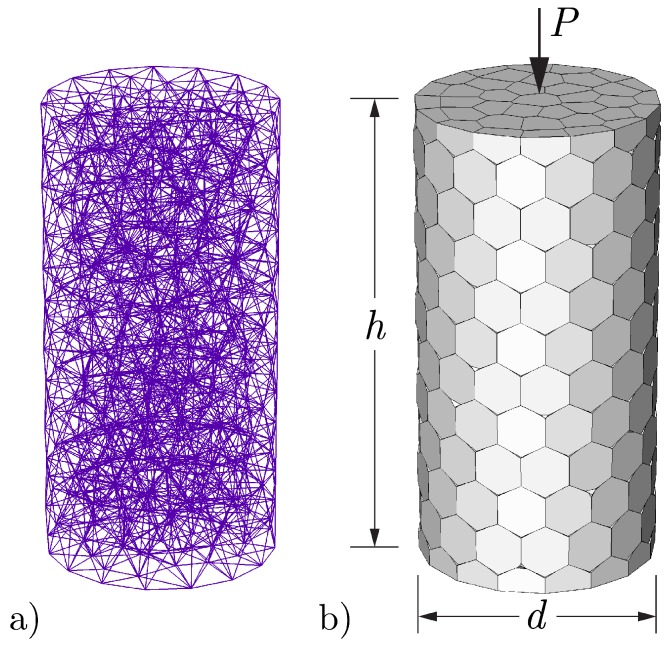
Discretization for creep test simulations: (**a**) lattice representation; and (**b**) volume rendering of cylindrical specimens.

For the creep simulations, the cylindrical specimens are discretized as shown in Figure 6. The cylinder dimensions of 100 mm by 200 mm differ from those of the experiment [75], but that is irrelevant for the simulation of basic creep of a material with uniform properties. A compressive load is applied uniformly over the top and bottom faces of the cylinder without lateral restraint. Each element of the lattice is a spring-dashpot construct that also accounts for hygral and thermal effects, as shown in Figure 5. At *t* = 0, the freshly cast concrete is assumed to be fully saturated (*h* = 1). The radial surface of the concrete exchanges heat with the environment (at *T* = 21 ${}^{\circ }$C, as recorded in the experiment) via a convective boundary condition. The cement composition is required as model input, as indicated in Section 2.3. For this simulation, a typical ASTM Type I cement was assumed [76]: $C_{3}S$ = 55%; $C_{2}S$ = 22%; $C_{3}A$ = 10%; $C_{4}\mathrm{AF}$ = 8%; and Blaine fineness = 365 m${}^{2}$/kg. A single set of creep parameter values has been used for the series of simulations: $q_{1}$ = 11.5 × 10${}^{-6}$/MPa; $q_{2}$ = 28 × 10${}^{-6}$/MPa; $q_{4}$ = 0.18 × 10${}^{-6}$/MPa; and $n_{\alpha }$ = 1.6.

**Figure 7:**
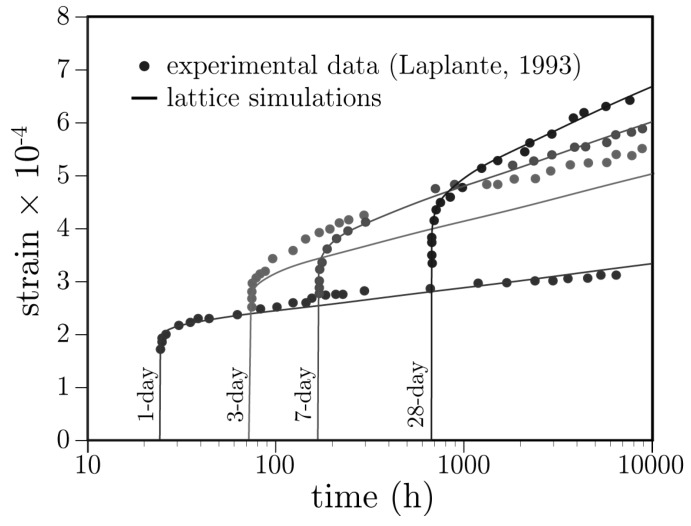
Basic creep curves for loading at different ages.

Comparisons of the model and experimental results are presented in Figure 7. The simulation results are reasonably accurate except for the case of 3-day loading, for which the simulated creep values are significantly less than the measured values. Creep associated with microprestress is activated by changes in temperature and internal humidity of the concrete [25]. Although moisture exchange with the environment is prevented by sealing, free water is consumed by the hydration process and such drying activates creep due to microprestress (i.e., the model simulates the effects of self-desiccation on creep).

**Figure 8:**
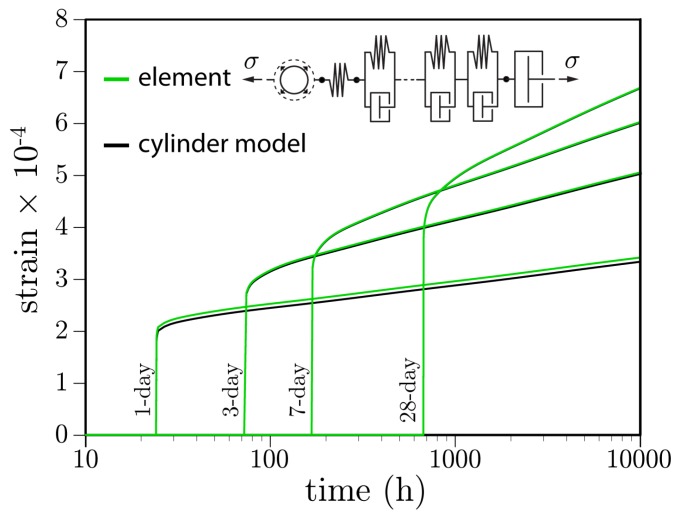
Basic creep curves produced from a single element and from a fully discretized cylindrical specimen.

Due to the lack of friction on the planes of load application, all material points are in a state of uniaxial compression. Neglecting the effects of non-uniform temperature within the specimen due to heat exchange with the environment, the cylinder model of Figure 6 should provide precisely the same results as a single element aligned with the loading direction. Such a comparison is given in Figure 8. The small difference between the 1-day loading curves is attributable to the accounting of heat of hydration in the cylinder model, whereas the single element was held at the ambient temperature of 21 ${}^{\circ }$C. Heat of hydration increases the degree of reaction, albeit only slightly for the case of a small cylindrical test specimen. At later ages of loading, the differences in degree of hydration are insignificant, such that the creep deformation curves are indistinguishable. These results highlight the ability of the irregular lattice to simulate uniaxial creep, using solidification and microprestress theory, without mesh bias.

### 3.2. Strength Development

**Figure 9:**
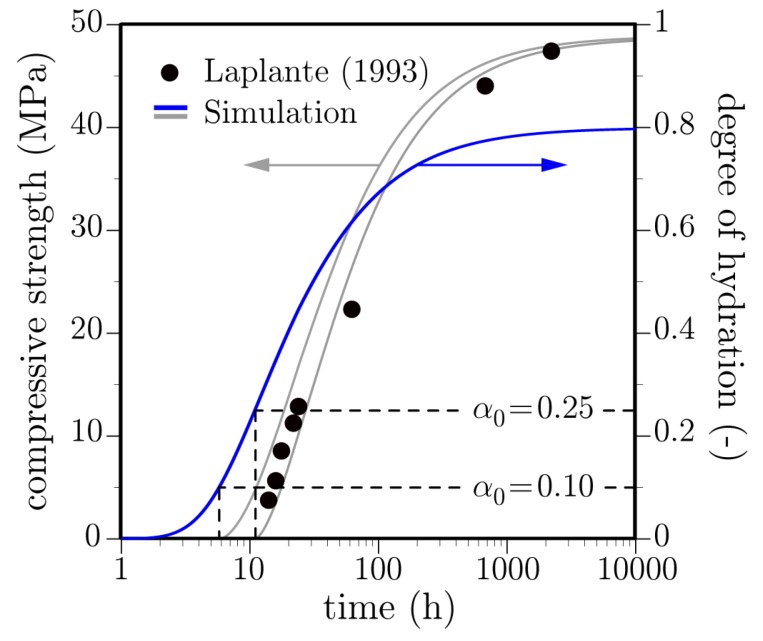
Strength development as a function of degree of hydration: dependence on setting parameter, $\alpha_{0}$.

For compressive strength development, Equation (28) becomes

(39) $$f_{c}\left(\alpha \right)=f_{c}\left(\alpha_{u}\right){\left(\frac{\alpha -\alpha_{0}}{\alpha_{u}-\alpha_{0}}\right)}^{\zeta }\;\;\;\mathrm{for}\;\;\;\alpha \geq \alpha_{0}$$

where $f_{c}\left(\alpha_{u}\right)$ is the asymptotic limit of compressive strength with degree of hydration. Herein, exponent *ζ* is assigned the typical value of 1.5 [41]. Figure 9 compares estimated strength values with those of the Laplante study, which was considered in the previous section. Strengthening commences for $\alpha \geq \alpha_{0}$. A typical range of $\alpha_{0}$ provides reasonably good estimates of strength development, which supports the validation of the hydration model.

### 3.3. Autogenous and Drying Shrinkage Tests

The series of tests of Yang et al. [77] are simulated. The test program involved the autogenous and drying shrinkage testing of blended cement concrete at three water-to-cementitious materials ratios: 0.25, 0.35, and 0.45. The binder consisted of ordinary portland cement and blast furnace slag at a 50:50 proportion. The blast furnace slag was finely ground, having a Blaine fineness of 600 m${}^{2}$/kg, which likely accentuated autogenous shrinkage. For each w/c value, both autogenous shrinkage and drying shrinkage tests were conducted using 100 × 100 × 400 mm${}^{3}$ prisms. When drying was considered, it was initiated at 1, 3, or 7 days. The 3-day and 7-day tests are simulated hereafter. Results from the 1-day drying experiments exhibit a qualitatively different trend that could not be captured using the same set of model parameters.

**Figure 10:**
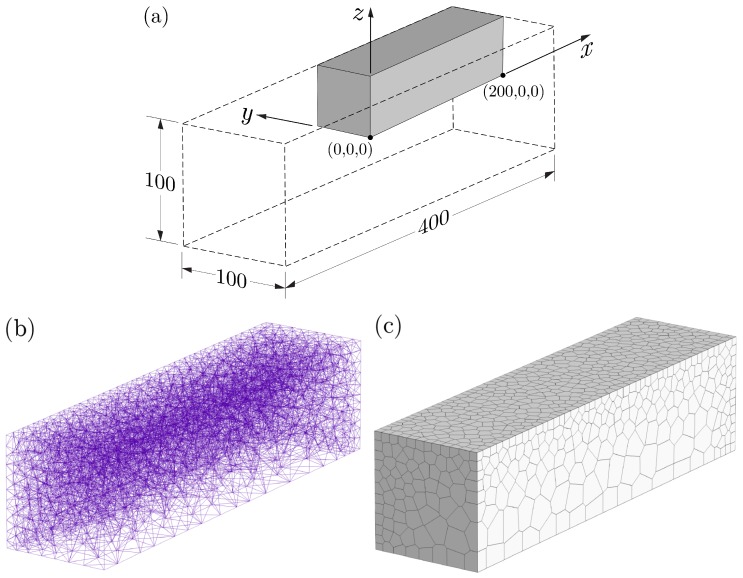
Discretization for shrinkage test simulations: (**a**) dimensions (in mm); (**b**) lattice representation; and (**c**) volume rendering of a symmetric portion of the prism specimens.

For the lattice modeling of the tests, symmetry conditions are exploited such that 1/8 of the prism volume is discretized (Figure 10). Heat and moisture exchanges occur across the exterior surfaces, but are prevented across the planes of symmetry. In addition, nodes along the planes of symmetry do not move normal to those planes of symmetry. Nodes were placed at ends of the prism, along the longitudinal axis, to serve as gage points for calculating longitudinal strain. Nodal density has been reduced within the core of the specimen where temperature and hygral gradients are relatively small.

Chemical composition of the portland cement fraction was not reported, so that values typical for Type I OPC have been used for the hydration model calculations. Humidity change due to self-desiccation and concrete diffusivity are represented by Equations (16) through (19) with the parameter values given in Table 3. The large $h_{su}$ values are due, in part, to the high volumes of finely ground slag in the concrete mixtures.

**Figure 11:**
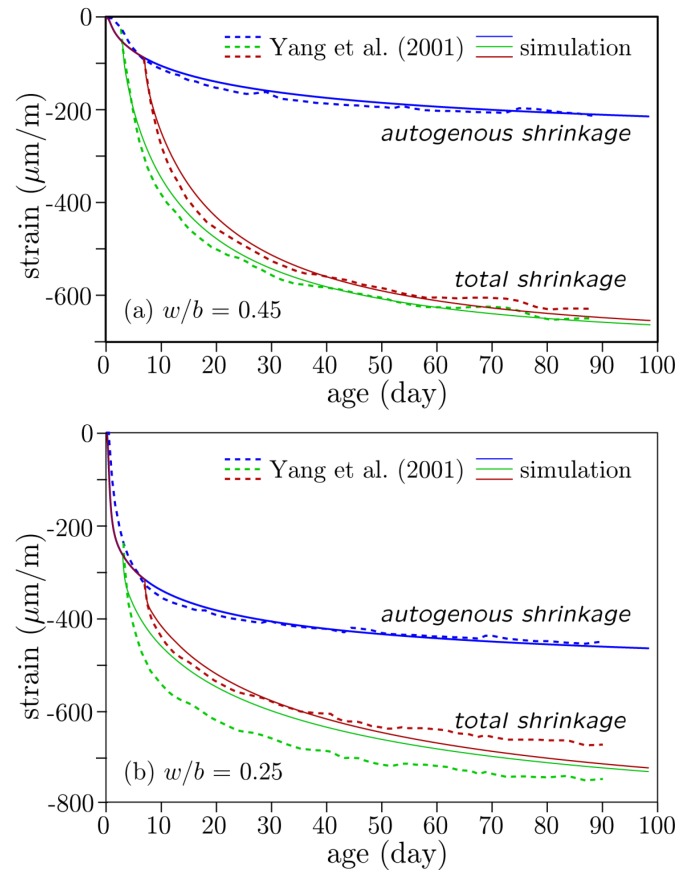
Autogenous and drying shrinkage simulations.

The simulated shrinkage strains are compared with the experimental values in Figure 11. The overall trends are captured well particularly for the autogenous shrinkage strain curves, which are relatively easy to fit. For the cases of drying, however, the experimental results exhibit sustained high rates of shrinkage in the first few days. That behavior can be simulated more precisely by increasing the diffusivity and/or hygral coefficient of the concrete, but then the ultimate shrinkage values are too large. The model is limited to the service range of loading (i.e., linear behavior, including creep) and does not account for the nonlinear effects of microcracking and creep damage under sustained high loading. Ultimately, those effects need to be incorporated into the analyses. The fitting exercise presented here should be understood in that context.

### 3.4. Analysis of Concrete Bridge Decks

As part of a Caltrans sponsored project [78], the early-age behavior of concrete bridge decks was studied through their instrumentation and data acquisition for a period of time after concrete casting. Sensors positioned within the freshly cast concrete monitored temperature, internal relative humidity, and strain. Along with these sensor readings, ambient temperature, relative humidity, and wind speed were recorded on-site by a weather station. Observations of cracking behavior were also made. Herein, simulated temperatures are compared with such field measurements for the Markham Ravine Bridge on Route CA-55 near Lincoln, California. In addition, concrete cylinders and prisms were cast on-site for measuring strength and drying shrinkage, respectively. Simulations of these specimens, along with the deck temperatures, serve to calibrate the lattice model for the parametric study of deck cracking potential in the next chapter.

#### 3.4.1. Model Definition

**Figure 12:**
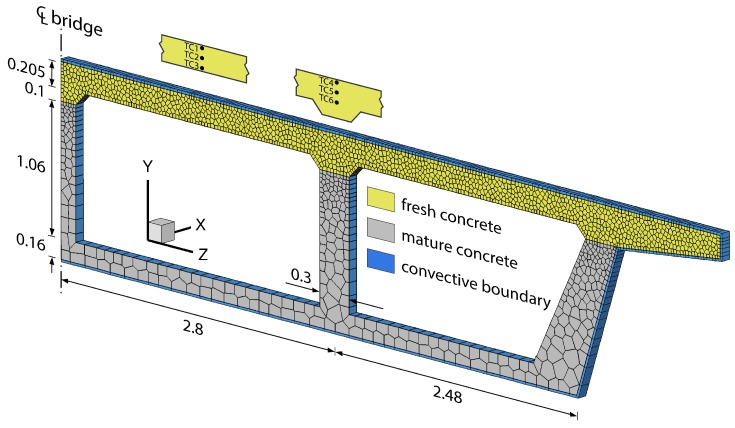
Lattice representation of symmetric portion of Markham Ravine bridge deck. The locations of thermocouples TC1, TC2, TC3 (midspan) and TC4, TC5, TC6 (over girder stem) are indicated. Dimensions are in meters.

**Figure 13:**
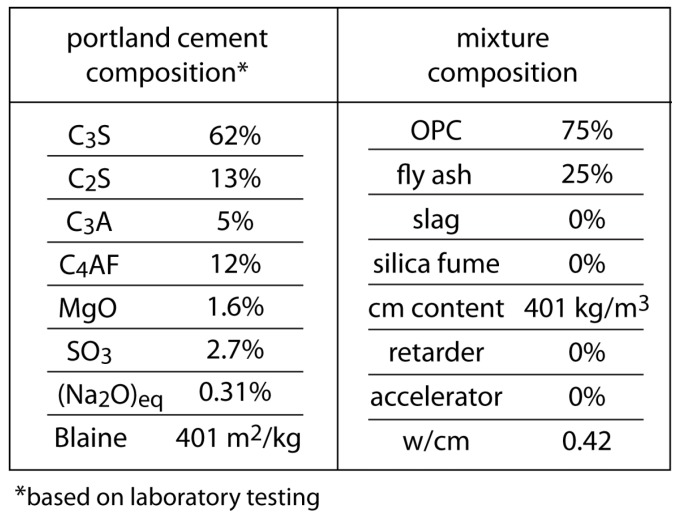
Input parameters for hydration model.

Discretization of the bridge deck/girder system is shown in Figure 12. Nodes are placed at the thermocouple locations, as indicated in the figure. Concrete forming the soffit and girder stems is assumed to be mature. Simulation of cementitious materials hydration is limited to the freshly cast deck. Inputs to the hydration model are indicated in Figure 13. Whereas the computational framework is three-dimensional, the simulations presented herein are for planar characterizations of the structure. The effects of reinforcing steel on the thermal and mechanical components of the analyses have not been included. Whereas the restraint provided by reinforcing steel reduces the free shrinkage of concrete, the modeling of this effect in a planar analysis framework is not straightforward. Reinforcement elements can be readily incorporated into this form of lattice model [79] and we plan to do so after extending the analysis to three dimensions. Boundary conditions for the thermal, hygral, and mechanical analyses are described in the following subsections.

**Thermal analysis inputs:** The initial temperature of the simulated, cast concrete was set to its measured value at the time of casting (19.5 ${}^{\circ }$C). The initial temperature of the simulated, mature concrete was set to the value measured at mid-height within one of the girder stems at the time of casting (14.9 ${}^{\circ }$C).

**Figure 14:**
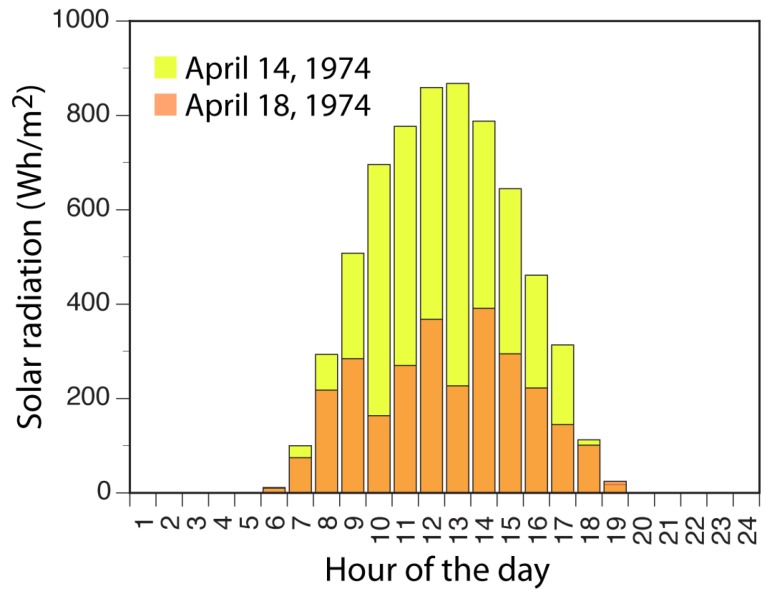
Solar radiation intensities for typical clear and overcast days.

Figure 14 shows typical measured solar radiation intensities for clear and overcast days for the geographic proximity and time of the year when casting occurred [80]. Along with an additional profile, representing broken cloud cover, these intensity plots are used as templates for the daily solar radiation input to the model.

One point of interest is the use of thermal/curing sheets on the cast surface. A combination of burlap and plastic membrane (Transgard 4000) was used to cover the bridge deck for the time interval 0.25≤t≤9 days, where *t* = 0 is the time of concrete casting. Placement of this curing medium modifies heat exchange with the environment.

(40) $$\begin{array}{cccc} q_{conv}^{\prime } & = & \eta_{1}\;q_{conv} & \\ q_{sun}^{\prime } & = & \eta_{2}\;q_{sun} & \mathrm{for}\;0.25\leq t\leq 9\;\mathrm{days}\\ q_{sky}^{\prime } & = & \eta_{3}\;q_{sky} \end{array}$$

in which $\eta_{1}$, $\eta_{2}$, and $\eta_{3}$ are reduction factors associated with each respective form of heat flux. According to experimental measurements [81], placement of a fabric/plastic membrane can reduce convective heat transfer by roughly a factor of 5 for the case of wind velocity *v* = 0 m/s, which results in $\eta_{1}$ = 0.2. The product sheet for Transgard 4000 indicates a light reflectance of 0.85. This is roughly twice the reflectance value of ordinary portland cement concrete, which ranges from about 0.34 to 0.48 [82], such that $\eta_{2}$ = 0.23. For lack of information, the same reduction factor was assumed for the sky radiation boundary condition (i.e., $\eta_{3}$ = 0.23).

The coefficient of convective heat transfer, $\Lambda_{T}$, was a function of measured wind speed [34]. Based on a series model [24], a modified value for $\Lambda_{T}$ was utilized to account for the presence of 1/2${}^{\prime \prime }$ (12.7 mm) plywood formwork along the lower face of the fresh concrete. The thermal conductivity of plywood was assumed to be 0.12 W/(m · K).

Measurements of the CTE of the hardened deck concrete provided $\beta_{Th}$ = 8.6 × 10${}^{-6}$/${}^{\circ }$C. Variation in CTE at early age was modeled using the expressions given in Table 2, in which *χ* = 3.5 based on measurements of Kada et al. [38] on various high-performance concrete mixtures. Similar large variations were observed for cement pastes and mortars in other studies [83].

**Hygral analysis inputs:** The ultimate value of relative humidity, associated with self-desiccation, was assumed to be $h_{su}$ = 0.9 with *s* = 3 governing the rate of self-desiccation according to Equation (16). The parameter values expressing hygral diffusivity, according to Equations (17) and (19), are: $\tilde{D_{0}}$ = 0.017 mm${}^{2}$/h; $\tilde{D_{1}}$ = 9 mm${}^{2}$/h, and *n* = 5.

The concrete surfaces bounded by plywood formwork are assumed to be sealed (i.e., no moisture exchange occurs between the concrete and plywood formwork.) While the curing sheeting is in place (0.25 d ≤t≤ 9 d), along with periodic wetting, no moisture is exchanged between the upper surface of the concrete deck and the environment. Otherwise, the exposed surface exchanges moisture with the environment according to Equation (20), using $\Lambda_{h}$ = 0.25 mm/h. Soon after finishing of the cast concrete, a light-colored membrane curing compound was sprayed on the deck surface. Based on laboratory measurements [84], this membrane is assumed to reduce convective transport across the surface by a factor of 2 (i.e., $q_{h}^{\prime }$ = 0.5 $q_{h}$). The hygral shrinkage coefficient $\beta_{h}$ = 0.0030 was calibrated with experimental measurements given in Section 3.4.2. A zero-flux condition was enforced across the plane of symmetry (*z* = 0).

**Mechanical analysis inputs:** The parameters for the solidification and microprestress modeling of concrete behavior are presented in Table 4. The creep parameter values were set according to the B4 model [85], based on the actual mixture composition. We have made no effort to distinguish between tensile creep, of interest herein, and compressive creep (even though tensile creep may be much larger than compressive creep for the same levels of applied stress [86]).

All stresses originate from thermal and hygral strains. Externally applied loads (e.g., due to construction activities) were not considered at this stage of the analysis. Elements spanning the cast deck and the mature concrete substrate were given properties of the substrate concrete. This is a reasonable approximation given the small element size and our relative lack of interest in behavior at that bi-material interface. A zero-displacement condition was enforced across the plane of symmetry (*z* = 0), along with a single support along that plane to prevent rigid-body motion in the vertical direction.

**Figure 15:**
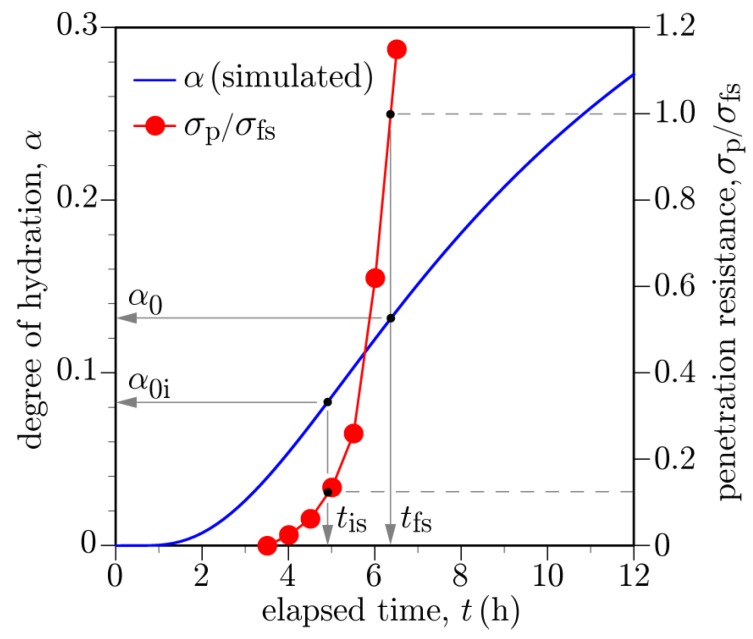
Determination of degrees of hydration at initial and final sets using recorded penetration resistance data [78].

The degree of hydration at concrete setting, $\alpha_{0}$, is required for the strength development model expressed by Equation (28). Within the test program associated with the Markham Ravine Bridge deck concrete, penetration resistance was measured according to ASTM C403 [87] to determine initial and final setting times, $t_{is}$ and $t_{fs}$, respectively. As per standard, mortar was extracted from the deck concrete and subjected to penetration testing. The results are shown in Figure 15, in which $\sigma_{p}$ is the measured penetration resistance and $\sigma_{fs}$ is the penetration resistance associated with final set. The prescribed resistance levels associated with initial and final set are 3.5 MPa (500 psi) and 27.6 MPa (4000 psi), respectively. The initial and final set times ($t_{is}$ = 4.9 h and $t_{fs}$ = 6.33 h) are determined by the intersections of these respective stress levels with the resistance curve.

To determine the degrees of hydration at initial and final set, a volume of mortar was simulated using the measured initial temperature of the mortar (18.89 ${}^{\circ }$C) and ambient temperature (21.1 ${}^{\circ }$C). For the thermal calculations, the coarse aggregates were removed within the hydration routine and in the calculation of specific heat capacity. The simulated degree of hydration is plotted as a function of time in Figure 15. The degrees of hydration associated with $t_{is}$ and $t_{fs}$ are $\alpha_{0i}$ = 0.085 and $\alpha_{0}$ = 0.13, respectively. Some studies suggest the development of the mechanical threshold (e.g., tensile resistance) of hardened concrete begins rather close to the time of initial setting [88]. Other sources use the point of final set to define the mechanical threshold [89,90]. The $\alpha_{0}$ value is used for strength development in the bridge deck simulations that follow. The influences of w/c and aggregate content on the mechanical threshold have been investigated via computational modeling [68].

#### 3.4.2. Simulation Results

**Figure 16:**
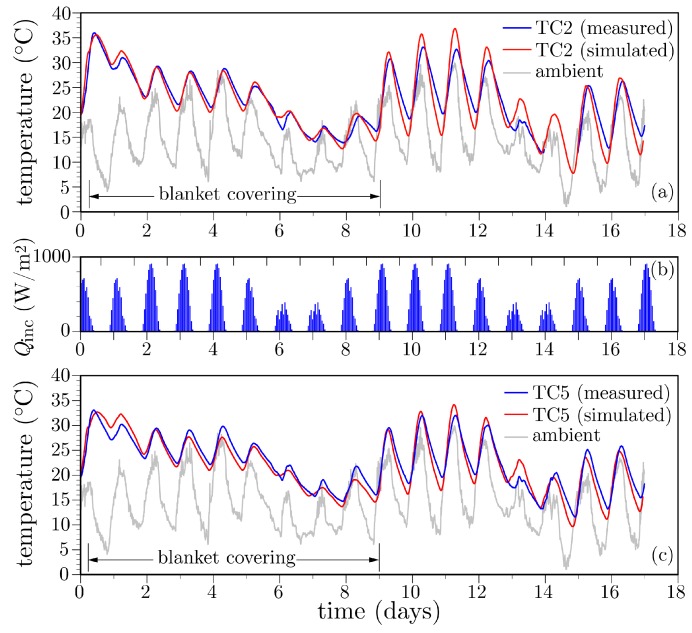
Temperature variation within the Markham Ravine Bridge deck: (**a**) mid-deck at location TC2; (**b**) solar radiation profiles selected for each day; and (**c**) above girder stem at location TC5.

**Figure 17:**
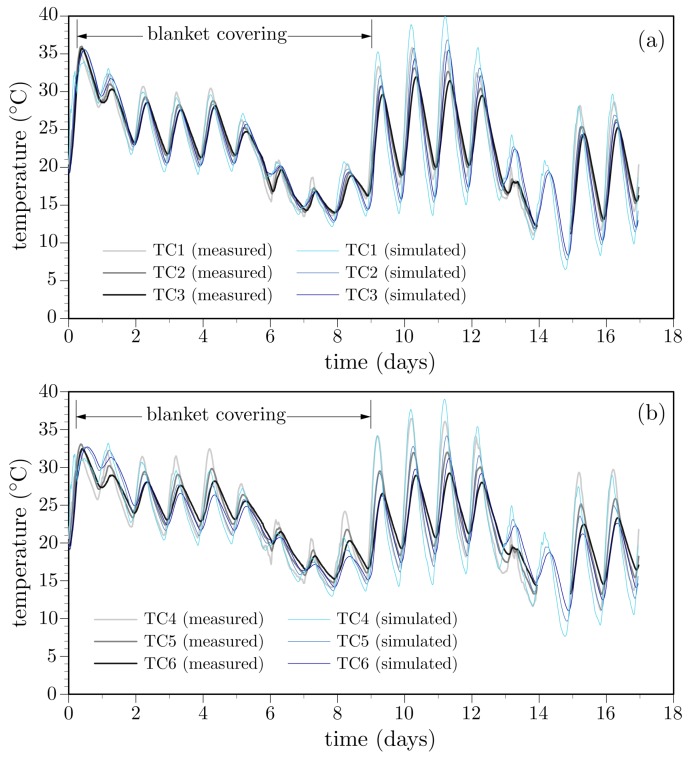
Measured and simulated temperatures at thermocouple locations.

**Deck temperature histories:** The simulated temperature histories are compared with the field measurements in Figure 16, for the case of thermocouples TC2 and TC5 located within the mid-deck region and above the girder stem, respectively. The recorded ambient temperature history is also plotted in the figures. Comparisons with the entire set of thermocouple readings are presented in Figure 17. Several comments can be made.

**Figure 18:**
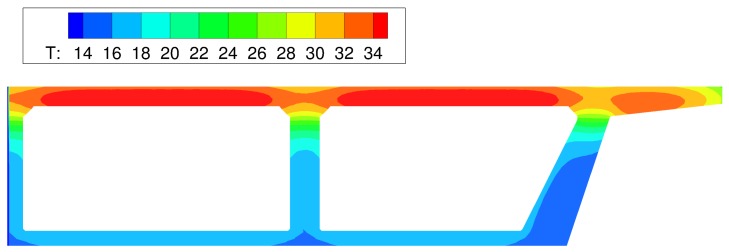
Simulated iso-contours of temperature (in ${}^{\circ }$C) at *t* = 10 h.

- The influence of environmental factors is evident from the oscillatory behavior of the temperature history recorded by each thermocouple. After the first day, locations closer to the surface exhibit larger temperature swings, whereas deeper locations are less affected by environmental changes. This meets expectations.
- Peak temperatures occur at about 10 h after concrete casting (Figure 18). The lower temperatures over the supporting girders are due to conduction of heat toward the cooler substrate concrete. Conversely, the insulative properties of the plywood formwork give rise to higher temperatures between the supporting girders. These temperatures are significantly higher than the ambient temperature. The differences between the ambient and measured deck temperatures largely diminish over the first two days after casting, in contrast to mass concrete applications in which large temperature differences can exist for several weeks. Despite the discrete, irregular discretization of the domain, the iso-contours of temperature do not exhibit artifacts associated with mesh bias.
- After removal of the curing sheets, not only do diurnal variations in deck temperature increase, but also the temperature gradient through the deck thickness tends to increase. The implications of the larger thermal gradients are discussed later.

**Figure 19:**
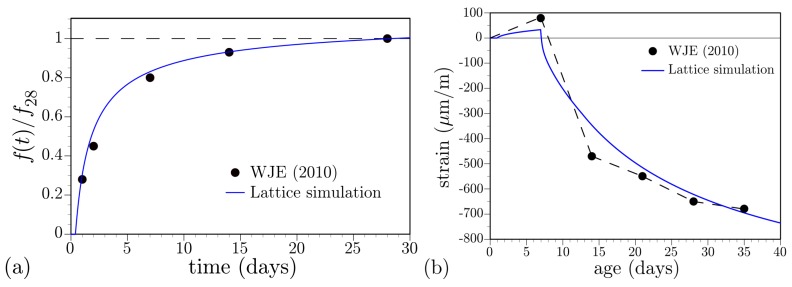
Property development in on-site cast and simulated specimens: (**a**) splitting tensile strength; and (**b**) shrinkage strain.

**Strength development:** Concrete cylinder specimens were cast on site and kept local to the bridge deck for 7 days, prior to laboratory storage and measurement of splitting tensile strength. Strength development was simulated using Equation (28), with $\alpha_{0}$ = 0.20, and the same lattice model adopted for the creep test simulations (Figure 6). Ambient temperature for the modeling exercise was constant and set equal to the average recorded temperature over the curing period. Figure 19a compares simulated strength development with the measured values, where the 28-day splitting tensile strength is used as a normalizing factor.

**Shrinkage development:** For measuring the drying shrinkage properties of the concrete mixture, prisms were cast on-site and transferred to the laboratory within 24 h, after which they were kept in a moist condition until exposure to a drying environment at *t* = 7 days. Testing was done according to ASTM C157, except for the 7 days of moist pre-conditioning according to Caltrans recommendations. The measured and simulated shrinkage strains are compared in Figure 19b. Initial expansion of the prisms was simulated by prescribing *h* = 0.97 as an initial condition prior to moist curing at 1 day. Whereas the sources of expansion may be due to other factors [91], this simulation of swelling demonstrates the expected workings of the model. As seen in the previous examples of autogenous and drying shrinkage, the shape of the simulated shrinkage curve does not conform to that of the experimental curve: the simulated rate of shrinkage over the first several days is lower. This could be remedied by increasing hygral diffusivity and/or the shrinkage coefficient, but the ultimate shrinkage strain would then be overestimated. Here, too, it is apparent that effects of microcracking need to be incorporated into the analyses.

## 4. Parametric Study

Through the preceding examples, basic workings of the thermal, hygral, and mechanical components of the model have been validated. The model is now used to assess the early-age cracking potential of the Markham Ravine Bridge. In particular, the relative importances of thermal and hygral contributions to cracking potential are evaluated for two design factors: (1) the use of curing media; and (2) restraint to deck movement. Cracking of the concrete is not simulated, except for the analysis results presented near the end of Section 4.2. The scope of this exercise is limited to planar analyses of the bridge deck using the lattice model shown in Figure 12. The implications of this simplification of the bridge deck configuration and boundary conditions are discussed later.

### 4.1. Curing Protocol

**Figure 20:**
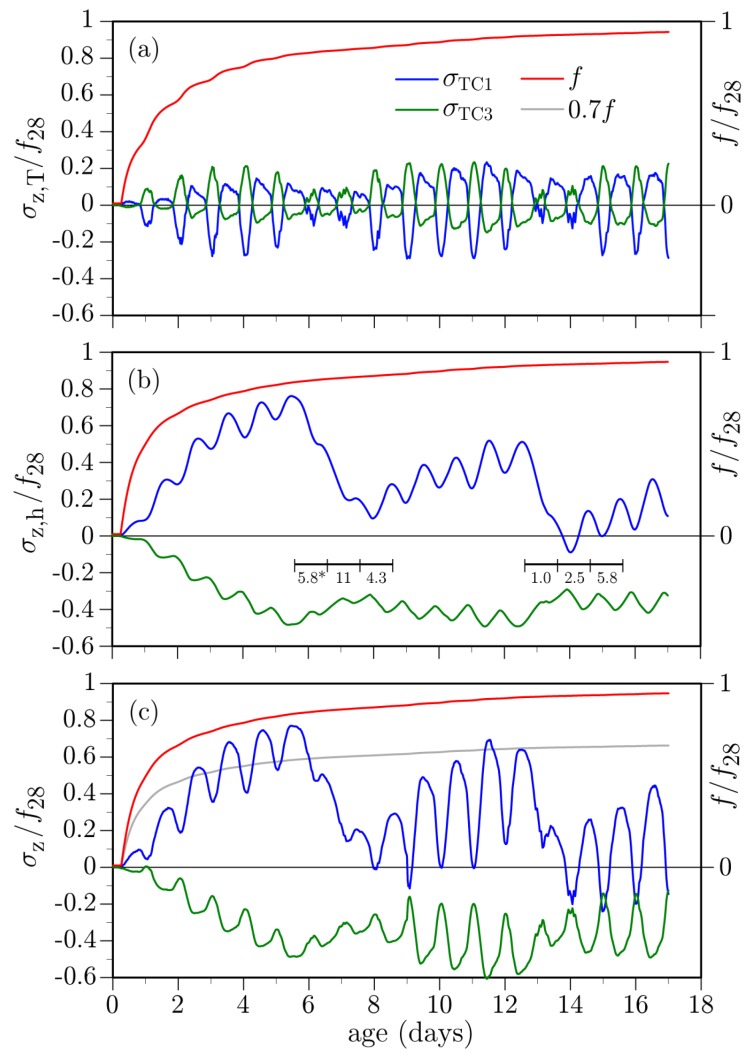
Simulated stresses at mid-deck location when the curing media is absent: (**a**) hygral stress component (* daily rainfall, in mm, recorded at the Carmichael 0.9 S meteorological station); (**b**) thermal stress component; and (**c**) total stress.

As noted above, curing compound and polymer/fabric sheets were applied on the concrete deck as part of the curing protocol. Concrete stresses at the mid-deck location, normalized by the 28-day tensile strength, are plotted in Figure 20 for the case when these curing media are absent. To compare the thermal and hygral stress contributions with their sum, the *z*-component of the stress tensor has been evaluated at the TC1 and TC3 thermocouple locations. These $\sigma_{Z}$ values are roughly equal to the principle tensile stress values at the same locations. The hygral and thermal contributions to total stress are isolated by setting $\beta_{T}$ = 0 and $\Lambda_{h}$ = 0, respectively.

As expected, exposure to the drying environment produces tension near the deck surface, due to the hygral gradient in the *y*-direction (Figure 20a). Location TC3 is in compression, as is much of the depth of the deck, in response to the surface tension. The oscillatory nature of the stress curves is due to diurnal variations in ambient relative humidity: relative humidity climbs at night due to decreasing temperature such that moisture intake and swelling occurs near the upper surface of the deck. The large drops in stress magnitudes beginning at about *t* = 6 and 13 days are due to rain events. Rainfall was not modeled directly, but rather captured through the relative humidity boundary condition. Sustained, large relative humidity values were measured on-site during those rain events. Based on simulated field testing of concrete, Asamoto et al. [92] have found that shrinkage of concrete is significantly reduced by rainfall and that continuous rainy days have such influence, even if the amount of precipitation is small.

Figure 20b plots the stresses associated with thermal effects. During daytime heating, the upper surface of the deck expands relative to the lower surface, producing compression at the TC1 location and tension at the TC3 location. This tendency is reversed during nighttime cooling. For either case, the tensile stresses are significantly smaller than those produced by hygral loading. For both the hygral and thermal loading cases, stress variations over the girder stem (at TC4 and TC6) are qualitatively similar, but slightly smaller in magnitude.

Tensile strength of the concrete at the TC1 location, as determined from the degree of cementitious material reaction (Equation (28)), is indicated in each plot within Figure 20. The evolution of tensile strength, scaled by a reduction factor of 0.7 (as discussed in Section 2.4.3), is also indicated in Figure 20c. The tensile stresses in Days 2, 3, and 4 cross over this curve, which suggests the occurrence of cracking.

**Figure 21:**
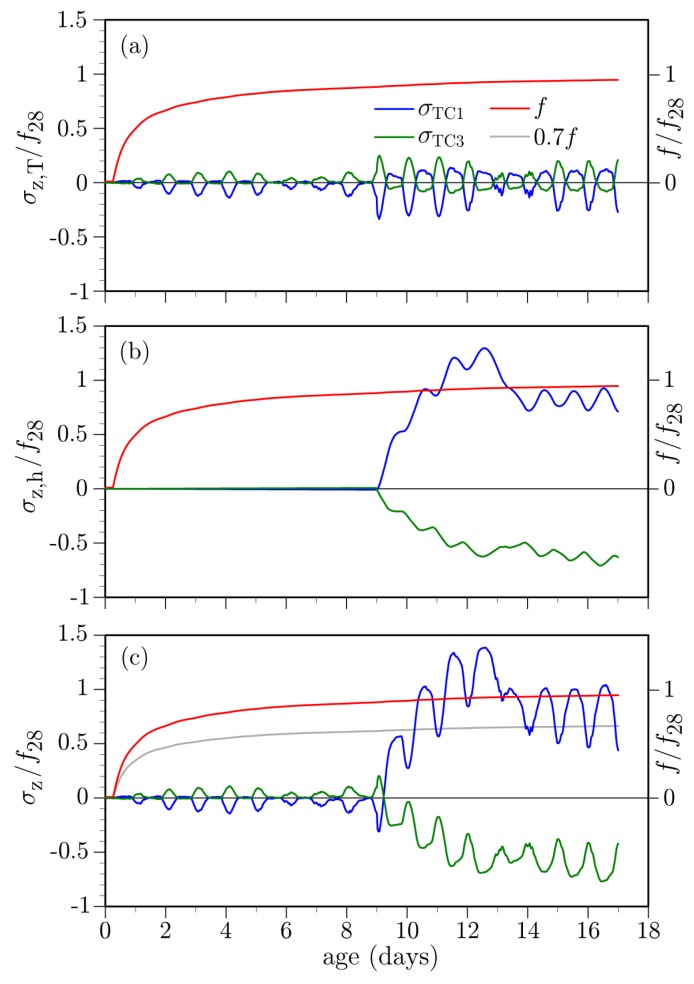
Simulated stresses at mid-deck location when the curing media is present during 0.25<t<9 days: (**a**) hygral stress component; (**b**) thermal stress component; and (**c**) total stress.

While in place, the curing sheets act to significantly reduce both the hygral and thermal stresses, as shown in Figure 21. Upon removal of the sheets, however, sudden drying of the deck surface from the nearly saturated state causes high tensile stresses. For this case, as well, the tensile stress values cross over the scaled tensile strength curve indicating the likelihood of cracking.

**Figure 22:**
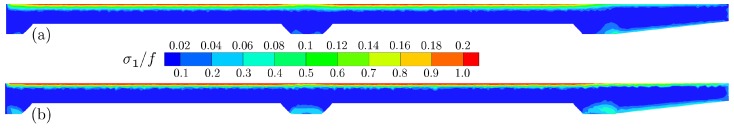
Spatial maps of cracking potential at *t* = 11.42 days (8 pm on day 11) for the case where the curing sheets were removed at *t* = 9 days: (**a**) thermal stress component; and (**b**) total stress.

Knowing the stress state and tensile strength at each nodal location, we can plot maps of cracking potential (i.e., $\sigma_{1}/f$, where $\sigma_{1}$ is principle tensile stress and *f* is the current tensile strength value at that location) over the computational domain. Figure 22 provides such plots for the thermal stress contributor and total stress at *t* = 11.42 days, which is near the time of maximum $\sigma_{1}/f$ for the TC1 location. To highlight thermal stresses, different scales are used for each plot. The highly stressed region extends about 40 mm (or 1/5 of the deck thickness) from the exposed surface. Similarly steep stress gradients have been found near the surface of concrete pavements [93] and bridge decks [9]. Cracking would tend to relieve stress along the drying surface and, due to the sharp gradient in stress, it appears that cracks would not propagate through the deck thickness. That hypothesis is evaluated using concrete fracture mechanics, as described later.

**Figure 23:**
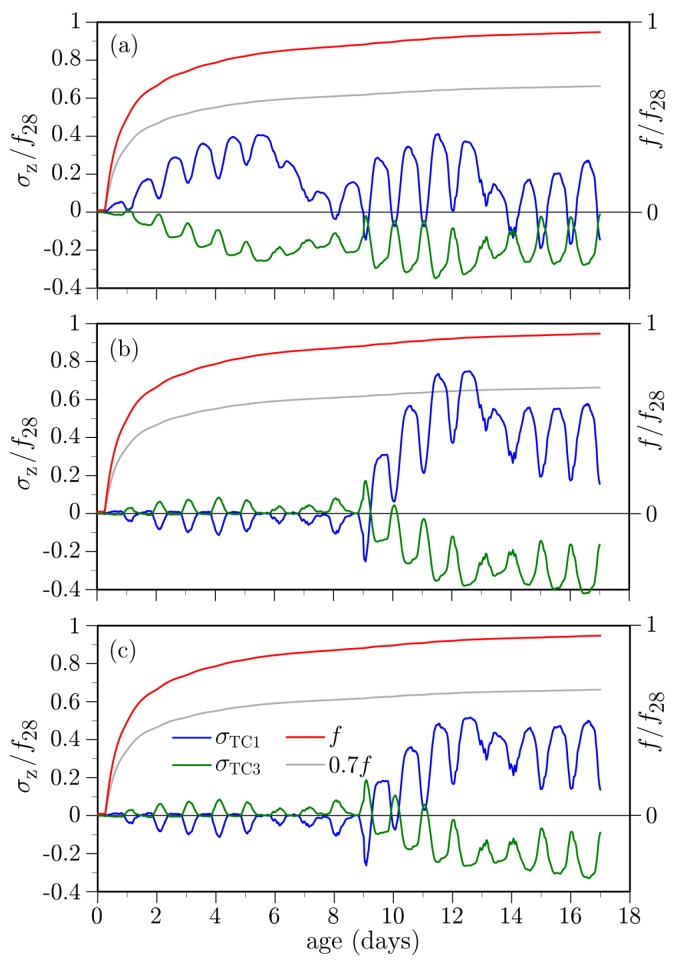
Simulated stresses at mid-deck location based on 28-day strain limit of 450 μm/m: (**a**) the curing sheet is absent; (**b**) curing sheet is present; and (**c**) curing sheet and curing compound used.

The strong potential for cracking indicated by Figure 20 through Figure 22 is accentuated by at least three factors: (1) the high 28-day shrinkage measured in the laboratory and used for the analyses (Figure 19b); (2) the low tensile strength of the test prisms used for this comparison; and (3) lack of consideration of membrane-type curing compound applied in the field study. These simulations have been rerun after: (1) scaling the drying shrinkage curve in Figure 19 to meet the 28-day limit of 450 *μ*m/m prescribed by several transportation agencies [94]; and (2) calculating the 28-day tensile strength according to $f_{t}=0.3{\left(f_{c}\right)}^{2/3}$, where $f_{c}$ is the measured 28-day compressive strength [89]. Based on these assumptions, the likelihood for cracking is greatly reduced, as shown in Figure 23a,b for each respective simulation case.

Furthermore, if a curing compound has been applied, the cracking potential is further reduced. For the simulation results presented in Figure 23c, application of the curing compound is assumed to reduce the hygral convective coefficient by 50%, as described in Section 3.4.1. Analyses of the effects of structural configuration, which follow, are based on the parameter combination corresponding to Figure 23b (in which the prism 28-day shrinkage strain is 450 μm/m and the tensile strength is based on the measured compressive strength).

### 4.2. Structural Configuration

**Figure 24:**
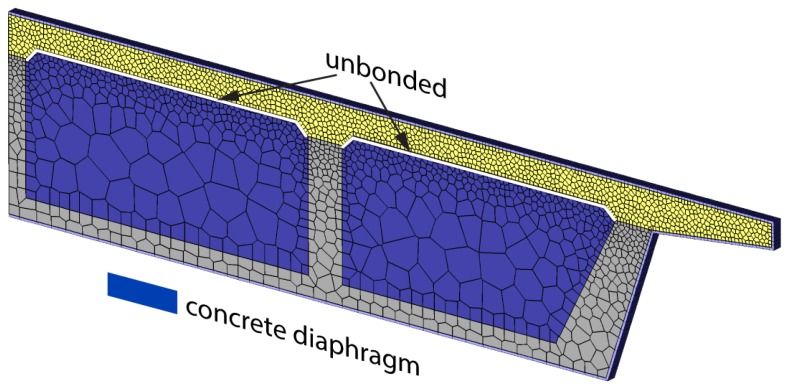
Discretization of box girder cross-section including cell volumes.

The structural configuration used for the previous analyses is representative of in-plane conditions away from supports or diaphragms placed along the span length. As a rough approximation of the conditions local to such stiffening elements, the cell volumes have been filled with concrete (Figure 24). The concrete within the cells is given the same properties as those of the soffit and girder stems. Otherwise, the following analyses retain the same set of parameter values used to produce the results in Figure 23b.

**Figure 25:**
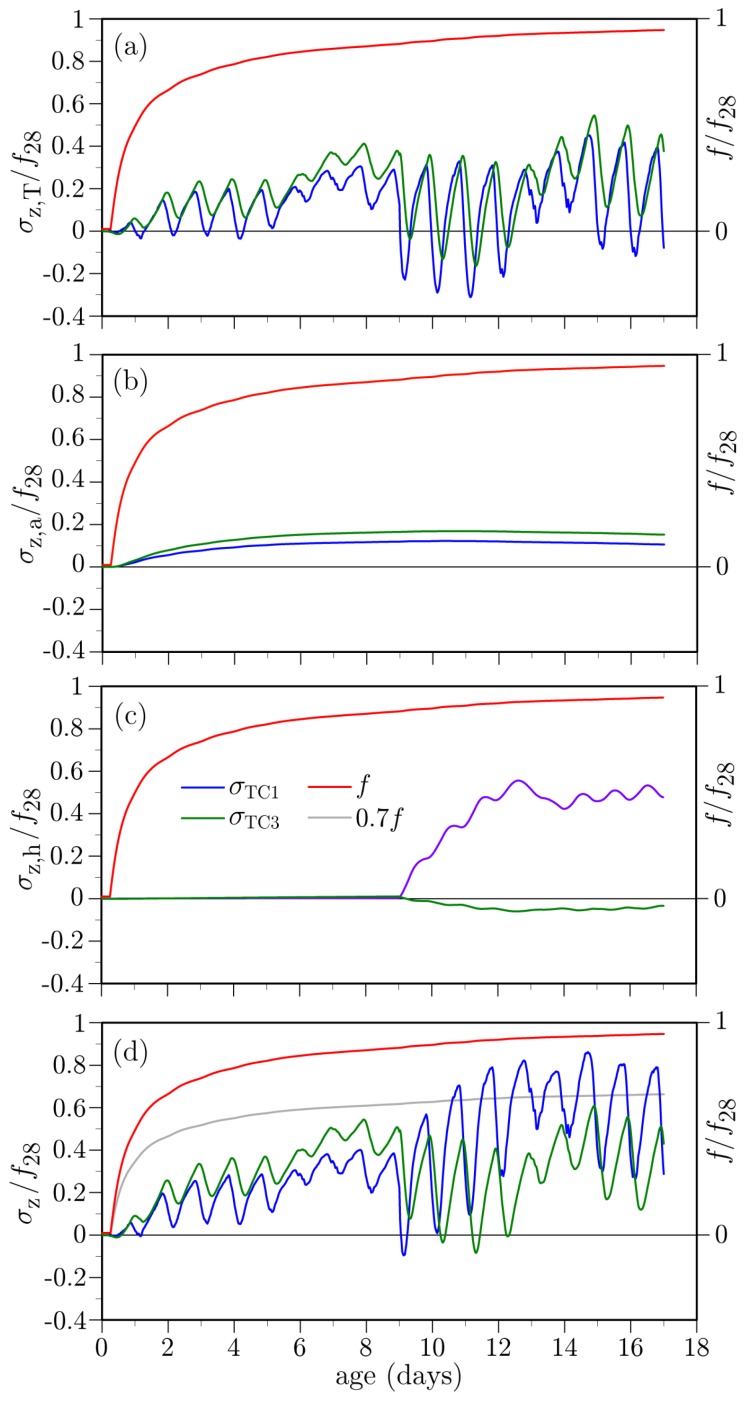
Simulated stresses at mid-deck location in the presence of constraint: (**a**) thermal stress component; (**b**) hygral stress component (due to self-desiccation); (**c**) hygral stress component (due to external drying); (**d**) total stress.

Stress values due to the thermal component are plotted in Figure 25a. The amplitudes of the diurnal variations are larger than for the unrestrained cases. Furthermore, the entire deck cross-section experiences tension, in contrast to the previous results where temperature gradients through the thickness produced mainly tension-compression couples. The increase in deck tension over the first several days corresponds to the overall cooling of the deck, as shown in Figure 17.

The deck stresses due to hygral effects are plotted in Figure 25b,c. The stresses due to autogenous shrinkage are significant, in contrast to the unrestrained cases where such stresses were negligible and therefore not plotted in the previous examples. In Figure 23b, for example, there is no upward drift into tension over the first 9 days when the curing sheets are in place. For the case of moisture loss to the environment (Figure 25c), the stress amplitudes are slightly higher than for the case without restraint. Otherwise, the results for the two cases are qualitatively similar.

Total stresses at the mid-span location, which include the thermal, autogenous, and drying shrinkage components, are plotted in Figure 25d. The total stresses are not a simple summation of the component values, since the autogenous and drying shrinkages are coupled through the dependence of hygral diffusivity on internal relative humidity (Equation (17)). Comparing with the case where restraint is absent (Figure 23b), the stress amplitudes plotted in Figure 25d are greater and the entire cross-section experiences tension.

**Figure 26:**
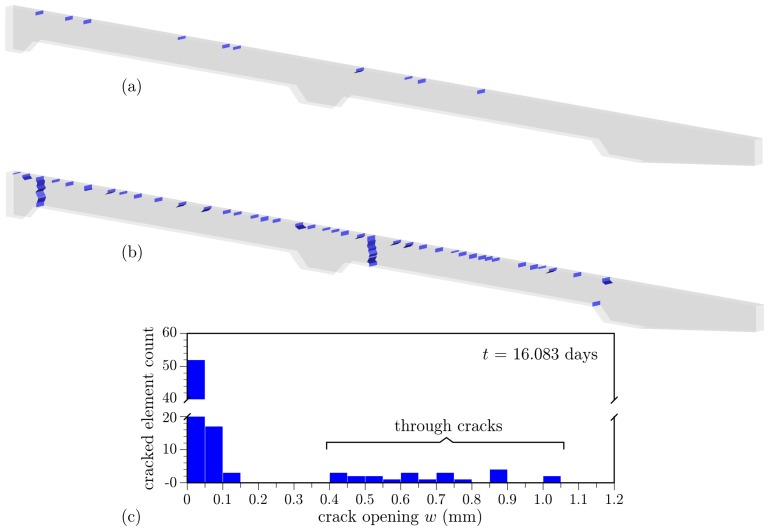
Simulated deck cracking: (**a**) without restraint; (**b**) with restraint associated with an internal diaphragm; and (**c**) crack opening histogram for the restrained case (at 12 pm on Day 16).

By allowing fracture to occur, according to the procedure outlined in Section 2.4.3, consequences of the restraint conditions become evident (Figure 26). When restraint is absent, the sharp humidity gradient (with secondary contributions from the thermal gradient) produces cracking near the drying surface. This has been observed in other studies, as well [95]. When the restraint is present, both autogenous shrinkage and global temperature change of the deck introduce tension over the deck cross-section. Crack density is higher and some cracks propagate through the deck cross-section, as shown in Figure 26b. Similar cracking behavior has been noted in field studies where concrete abutments provided fixity to the deck ends [9,96]. Transitions from diffuse distributed cracking to localized fracture are simulated simply and effectively, which is desirable for evaluating most concrete durability problems.

The surface cracking due to hygral and thermal gradients is more extensive than shown in Figure 26, which only depicts cracks larger than a prescribed threshold. Furthermore, the openings of such surface cracks are much smaller than those of the through cracks that form when restraint is present. This is seen in the histogram of cracked-element openings given in Figure 26c. As the drying front progresses with time, many of the surface cracks have closed and are therefore not counted in the histogram. The time of day also influences the opening counts. For the 12:00 pm counting shown in Figure 26c, solar heating of the top surface has reduced or closed some of the surface cracks. The scatter in openings for the through cracks, about an average opening of about 0.7 mm, is mainly due to flexure of the deck under thermal/hygral loading. Crack opening is proportional to depth within the deck cross-section.

Whereas these results indicate deck cracking would occur, the restraint condition associated with the internal diaphragm is an extreme case. Three-dimensional analysis of the deck is necessary to more accurately assess the effects of structural configuration on deck stresses. Furthermore, the effects of restraint and thermal straining in the longitudinal direction of the bridge are not captured by these planar analyses, even though such effects might contribute significantly to cracking potential.

## 5. Conclusions

This research pertains to modeling the early-age behavior of concrete materials with realistic structural and environmental boundary conditions. The discrete modeling approach, presented herein, is a novel assemblage of works of the authors and many other researchers. The relevant field quantities (temperature, relative humidity, and displacement) are represented by lattice models, which are defined by Delaunay/Voronoi tessellations of the computational domain. The simulations begin when the concrete materials are mixed. Based on a modeling of cementitious materials hydration, the simulations provide a spatial description of property (e.g., stiffness, strength) and stress development. This information can be used to assess the potential for early-age cracking. The following conclusions were deduced from this study:

1. This form of discrete model is capable of simulating the multi-field quantities associated with early-age concrete behavior, despite its discontinuous representation of the problem domain. There are no major disadvantages of this discrete approach with respect to continuum approaches, such as the finite element method. Advantages of this form of discrete model include its simplicity and adeptness at simulating the transition from diffuse damage to localized cracking.
2. Stress-to-strength ratio is lacking as a practical measure of cracking potential. Sharp hygral or thermal gradients near exposed surfaces typically cause high stresses, which is indicative of cracking. However, the cracking may only be superficial. Knowledge of the stress conditions through the structural cross-section is also necessary for evaluating the severity and potential consequences of cracking. In this sense, numerical modeling nicely complements knowledge gained by laboratory testing and field observations.
3. Structural configuration plays a key role in determining the magnitude and distribution of stresses caused by volume instabilities of the concrete material. Under largely restrained conditions, both thermal and hygral effects were found to be primary contributors to cracking potential, leading to crack propagation through the depth of the deck. Three-dimensional simulations are needed to assess the influence of longitudinal restraints, thermal flexing of the mature concrete girders, and the effects of reinforcing bars.
4. Realistic simulations of the early-age behavior of structural concrete require a wealth of information regarding the material constituents, production/curing processes, and structural and environmental boundary conditions. As shown by the parametric studies conducted herein, cracking potential is sensitive to input quantities that are typically not well defined, especially in field applications. Ultimately, the assessment of cracking potential needs to be cast in a probabilistic framework that accounts for uncertainties in the various inputs to the modeling effort.

## Figures and Tables

**Table 1:** Design parameters affecting early-age cracking of bridge decks.

Primary Category	Subcategory
Materials composition/proportioning	cementitious materials blend
	admixtures
	water content
	aggregate
	fiber reinforcement
Environmental boundary conditions	heat exchange: conduction, convection, radiation
	moisture exchange
	contaminant exposure
Processing and curing	concrete temperature at placement
	time of placement
	methods of consolidation
	curing
Structural boundary conditions	girder spacing and restraint conditions
	deck depth
	reinforcing steel
	anticipated loading

**Table 2:** Modeling of coefficient of thermal expansion.

$\beta_{T}/\beta_{\mathrm{Th}}$	Hydration Degree Interval
*χ*	$\;\alpha \leq \alpha_{0}$
$\chi -\left(\chi -1\right)\left(\alpha /\alpha_{0}-1\right)/\left(\varphi -1\right)$	$\alpha_{0}<\alpha <\varphi \alpha_{0}$
1	$\;\alpha \geq \varphi \alpha_{0}$

**Table 3:** Parameter settings for simulations of autogenous and drying shrinkage.

Parameter	w/c = 0.25	w/c = 0.45
$\tilde{D_{0}}$	0.017 mm${}^{2}$/h	0.017 mm${}^{2}$/h
$\tilde{D_{1}}$	9 mm${}^{2}$/h	9 mm${}^{2}$/h
*n*	6	5
$h_{su}$	0.76	0.88
*s*	1.4	3.0
$\Lambda_{h}$	0.25 mm/h	0.25 mm/h
$\beta_{h}$	0.0025	0.0021

**Table 4:** Parameter values associated with creep and stiffness development.

Parameter	Value *
$q_{1}$	29.0 × 10${}^{-6}$/MPa
$q_{2}$	69.9 × 10${}^{-6}$/MPa
$q_{4}$	5.50 × 10${}^{-6}$/MPa
$n_{\alpha }$	2.2
$\kappa_{0}$	0.01/(MPa · d)
$\kappa_{1}$	5 MPa/K

* $q_{1}$, $q_{2}$, and $q_{4}$ values are based on the B4 model [85] for $E_{28}$ = 24.1 GPa; w/c = 0.42; a/c = 6.13; and 25% replacement of normal cement with fly ash.

## Abbreviations

The following symbols are used in this manuscript:

**Symbol**	**Definition**
$h_{ij}$	Euclidean distance between nodes *i* and *j* [m]
*t*	time [s, h, d]
$A_{ij}$	area of Voronoi facet common to nodes *i* and *j* [m${}^{2}$]
Thermal analysis	
a/c	aggregate to cementitious materials ratio [kg/kg]
*c*	cementitious materials content [kg/m${}^{3}$]
$c_{p}$	specific heat capacity [J/(kg · K)]
$q_{conv}$, $q_{conv}^{\prime }$	heat flux due to convection and its modified value [W/m${}^{2}$]
$q_{sun}$, $q_{sun}^{\prime }$	heat flux due to solar radiation and its modified value [W/m${}^{2}$]
$q_{sky}$, $q_{sky}^{\prime }$	heat flux due to radiation and its modified value [W/m${}^{2}$]
$t_{e}$	concrete equivalent age [h]
w/c	water-to-cementitious materials ratio [kg/kg]
*D*	thermal diffusivity [m${}^{2}$/s]
$E_{a}$	apparent activation energy [J/mol]
*H*	cumulative amount of heat produced by hydration [J/kg]
$H_{cem}$	heat of hydration of cement [J/kg]
$H_{u}$	total heat available for reaction [J/kg]
*Q*	rate of heat production by cementitious materials hydration [J/(kg · s)]
*R*	universal gas constant [J/(mol · K)]
*T*	temperature [${}^{\circ }$C]
$T_{a}$, $T_{c}$, $T_{r}$, $T_{s}$	ambient, concrete, reference, and surface temperatures [${}^{\circ }$C]
$T_{sky}$	sky temperature [${}^{\circ }$C]
*α*, $\alpha_{u}$	degree of cementitious materials hydration and its ultimate value
*β*, *τ*	hydration model parameters
$\beta_{T}$, $\beta_{Th}$	coefficient of thermal expansion and its long-term value [1/${}^{\circ }$C]
$\gamma_{abs}$	solar absorptivity of concrete
*ϵ*	emissivity of concrete
$\eta_{1}$, $\eta_{2}$, $\eta_{3}$	heat flux reduction factors
*λ*	thermal conductivity [W/(m · K)]
*ρ*	mass density of concrete [kg/m${}^{3}$]
*σ*	Stefan-Boltzmann constant [W/(m${}^{2}$ · K${}^{4}$)]
*χ*	magnifying factor for coefficient of thermal expansion at early-ages
$\Lambda_{T}$	coefficient of convective heat transfer [W/(m${}^{2}$ · K)]
$M_{e}$, M	elemental and system capacity matrices
$K_{e}$, K	elemental and system conductivity matrices
Hygral analysis	
*h*	relative humidity [-]
$h_{a}$	ambient relative humidity [-]
$h_{s}$, $h_{su}$	relative humidity associated with self-desiccation and its ultimate value [-]
*n*	parameter relating hygral diffusivity and relative humidity
$q_{h}$, $q_{h}^{\prime }$	moisture flux due to convection and its modified value [m/s]
*s*	parameter relating humidity decrease and self-desiccation
$D_{0}$, $\tilde{D_{0}}$	diffusivity values associated with fully dried material [m${}^{2}$/s]
$D_{1}$, $\tilde{D_{1}}$	diffusivity values associated with saturated material [m${}^{2}$/s]
$D_{h}$	hygral diffusivity [m${}^{2}$/s]
$\beta_{h}$	hygral shrinkage coefficient
$\Lambda_{h}$	hygral convection coefficient [m/s]
Mechanical analysis	
*b*	element thickness [m]
$f_{c}$, $f_{cu}$	compressive strength and its asymptotic limit [MPa]
*f*	tensile strength [MPa]
$k_{n},k_{s},k_{t}$	stiffness coefficients of the uniaxial springs [N/m]
$k_{\phi x},k_{\phi y},k_{\phi z}$	stiffness coefficients of the rotational springs [N · m]
$n_{\alpha }$	parameter relating rate of solidification to degree of hydration
$q_{1}$, $q_{2}$, $q_{4}$	instantaneous elastic, viscoelastic, and viscous strain parameters [1/MPa]
$t_{0}$	age of loading [h]
$t_{e}$	equivalent age [h]
$t_{r}$	reduced time representing the thermal-hygral effects on creep [h]
$t_{is}$, $t_{fs}$	times of initial and final concrete setting [h]
*w*	crack opening [m]
$w_{c}$	traction-free crack opening [m]
$A_{ij}^{P}$	projected area of element facet [m${}^{2}$]
*E*	elastic modulus of concrete [MPa]
$E_{v}$, $E_{S}$	activation energies for creep and microprestress processes [J/mol]
$F_{n}$, $F_{s}$, $F_{t}$	spring-set forces in the normal and two tangential directions [N]
$I_{11},I_{22}$, $J_{p}$	principal planar and polar second moments of facet area [m${}^{4}$]
D	material constitutive matrix
$K_{e}$, K	elemental and global stiffness matrices
$\alpha_{0i}$ , $\alpha_{0}$	degrees of hydration at initial and final setting
φ	coefficient governing concrete setting behavior
*γ*	viscoelastic microstrain
$\varepsilon^{h}$, $\varepsilon^{T}$	hygral and thermal strains
$\varepsilon^{i}$, $\varepsilon^{v}$, $\varepsilon^{f}$	instantaneous elastic, viscoelastic, and viscous flow strains
*ζ*	coefficient governing material property development
*η*	effective microscopic viscosity [MPa/s]
*κ*	stress-to-strength ratio
$\kappa_{0}$	microprestress model parameter [1/(MPa · d)]
$\kappa_{1}$	microprestress model parameter [MPa/K]
*ν*	Poisson’s ratio
$\theta_{R}$	inclination of tensile force resultant relative to the element axis
$\vartheta_{1}$, $\vartheta_{2}$	parameters defining the break-point in the tension softening relation
*σ*	axial stress [MPa]
$\sigma_{R}$	stress resultant [MPa]
$\sigma_{p}$, $\sigma_{fs}$	penetration resistance and its value at final setting [MPa]
$\psi ,\psi_{S}$	reduced time coefficients for creep and microprestress processes
*ξ*	factor relating normal and shear spring stiffnesses
*ω*	degree of damage
Φ	non-aging micro-compliance function [1/MPa]
